# Zinc from an Essential Element to an Antiparasitic Therapeutic Agent

**DOI:** 10.1021/acsomega.4c07331

**Published:** 2025-01-17

**Authors:** Maribel Navarro, Luana Vanessa Daniel, Legna Colina-Vegas, Gonzalo Visbal

**Affiliations:** † Laboratório de Químicas Bioinorgânica e Catalise (LaQBIC), Departamento de Química, Instituto de Ciências Exatas, Universidade Federal de Juiz de Fora Juiz de Fora, Juiz de Fora 36036-900, Brazil; ‡ Instituto de Química, Universidade Federal do Rio Grande do Sul (UFRGS), Porto Alegre 91501-970, Brazil; § Laboratório de Ácidos Nucleicos (Laban), Coordenação Geral de Biologia (Cobio), Diretoria de Metrologia, Científica e Industrial, DIMCI, Instituto Nacional de Metrologia, Qualidade e Tecnologia (INMETRO), Rio de Janeiro 25250-020, Brazil

## Abstract

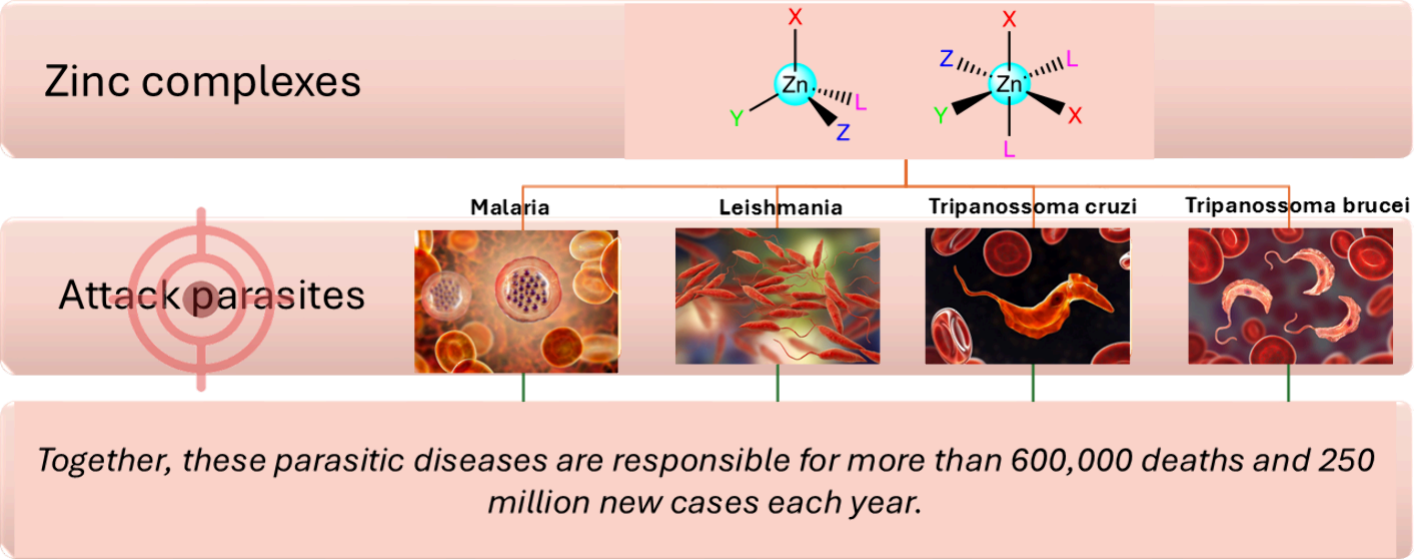

Tropical parasitic diseases affect millions of people around the world, particularly in poor countries. The human parasitic diseases that will be covered in this review are malaria and neglected diseases, such as leishmaniasis, Chagas disease, and African trypanosomiasis. The current treatments for these diseases present several problems, such as the development of drug resistance, very limited drugs available in the clinic, significant side effects of the drugs, and a long treatment period. For these reasons, there is an urgent need to develop new chemotherapeutics to eradicate or eliminate these diseases. Zinc-based drugs against parasitic diseases could be an alternative therapy to overcome the difficulties of the approved metallodrugs as antiparasitic agents. Zinc-based drugs are becoming an exciting field of research because zinc is an essential element that can lead to the development of multitarget antiparasitic agents, which are reviewed here.

## 1 Introduction

Zinc is an essential element and is the second most abundant trace metal in the body. It is involved in hundreds of different cellular processes as a cofactor of various metalloenzymes, such as the enzyme superoxide dismutase, in DNA and protein synthesis, intracellular signaling and division, and enzyme activity. Its role can be structural or cocatalytic.^1,2^

**Figure 1:**
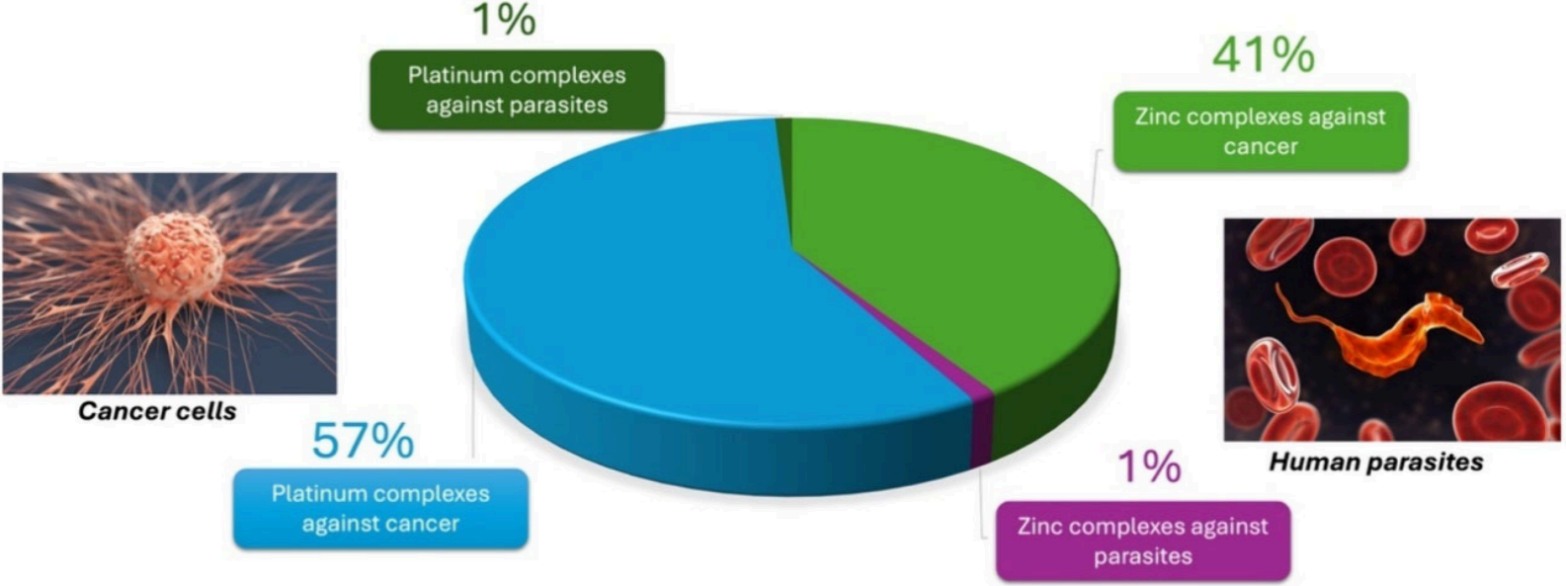
Number of articles in Web of Science on 04/24/2024, on the topics of zinc and platinum complexes against neglected diseases or cancer (2000–2023). Total number of articles found: 9786. They are distributed as follows: zinc complexes against parasites, 111; zinc complexes against cancer, 3986; platinum complexes against parasites, 95; platinum complexes against cancer, 5594.

It is of paramount importance to understand the role of Zn in vital biological functions, ranging from cellular zinc metabolism and zinc signaling and its effect on the progression of various diseases. However, zinc-based drugs have received very little attention compared to other metal-based drugs in clinical use, such as platinum (cisplatin, carboplatin, oxaliplatin, nedaplatin, lobaplatin, heptaplatin), which successfully treats several types of cancer worldwide, or gold (auranofin, solganol, mychrosine) and silver (silver sulfadiazine).^3,4^ There are some reports of zinc oxide or salts used in medicine: zinc chloride for its antiseptic properties and zinc sulfate to induce vomiting. Since the 1930s, zinc pyrithione has been used for topical treatment of skin or hair damaged by fungal or bacterial infections. Various zinc coordination compounds are currently being investigated for several applications in radioprotection, tumor photosensitizers, antidiabetic agents, anticonvulsants, anti-inflammatory agents, and agents with antimicrobial, antioxidant, and antitumor/antiproliferative properties.^5−7^ Research related to the antiparasitic activity of zinc complexes has been scarce compared to the number of cancer studies of platinum, even zinc, as shown in Figure 1.

Zinc in coordination compounds exists only in the +2 oxidation state, which corresponds to a filled 3d-shell ([Ar]d^10^ electron configuration). They are diamagnetic compounds and do not participate in chemical redox processes. Zn(II) complexes have no geometric preference based on LFSE (ligand field stabilization energy). Zn(II) is borderline HSAB (hard and soft acids and bases (HSAB)). This principle predicts that hard acids (metal) prefer hard bases (ligand), soft acids prefer soft bases, and borderline acids, like Zn(II), prefer borderline bases that have intermediate characters such as nitrogen, oxygen, and also sulfur-containing ligands. The coordination sphere and geometry vary, depending on the ligand size and charge density. The tetrahedral and octahedral geometries are the most common; however, polyhedral complexes are also reported (tetrahedral, trigonal bipyramidal, octahedral, etc.). Zn complexes can also exist in dimer and polymer structures.^8^ As several ligands have an affinity to zinc, mixed ligand Zn(II) complexes can be formed with ligands acting as actors or auxiliary ligands. In the medicinal field, these ligands can be involved in target recognition or interfere with biochemical pathways. Ligands can be atoms, ions, or molecules (neutral, cationic, or anionic) depending on the drug development approach. The interactions between the zinc and the ligands often follow Pearson’s hard–soft acids–bases theory and the thermodynamic and kinetic properties are influenced by the ligand exchange reactions.^1,2,5,6^ These Zn(II) complexes can transform (ligand exchange) before reaching a desirable biological target. They act as a prodrug in the cell.^1,2,5,6^

Parasitic infections are diseases caused by parasites living and multiplying in the human body. These microorganisms require the host to obtain the nutrients necessary for their survival. Millions of people are infected around the world every year. There are three main types of parasites that cause infections in humans: protozoa, helminths, and ectoparasites. The focus of this review is on diseases caused by protozoa, particularly those that can be transmitted through the blood, such as malaria and trypanosomatid diseases: leishmaniasis, African trypanosomiasis, and Chagas disease. These diseases have been afflicting mankind because there is no vaccine or medical control to prevent them. Some progress has been made in vector control, which was the main method of prevention for decades and is still effective today.^9^ WHO recommends a coordinated approach to vector control, that includes a rational decision-making process to optimize the use of available resources to improve the efficiency, environmental impact, and sustainability of disease control.^10^ The currently available treatments are very limited and have serious side effects. We will also review current research using zinc-based drugs as an approach to develop novel chemotherapeutic agents to treat these parasitic infections.

## 2 Zinc Complexes against Malaria Parasites

Humanity has been suffering from malaria for centuries, and this disease is considered to be one of the deadliest and most devastating parasitic diseases. It remains a major threat to global public health. According to the World Health Organization (WHO),^11^ in 2022, there were an estimated 249 million cases, resulting in 608,000 deaths worldwide. African countries are the most affected by malaria infection, accounting for 95% of the world’s deaths, mostly young children. The causative agents of malaria are single-celled protozoan parasites of the genus *Plasmodium*. Over 200 different *Plasmodium* (*P.*) species have been described. The ones that infect humans and cause malaria are *P. falciparum*, *P. vivax*, *P. malariae*, *P. ovale*, and *P. knowlesi*. All species of these *Plasmodium* parasites have similar life cycles. Gaining an understanding of how the *Plasmodium* parasite survives and thrives took several years. The cycle is divided into two parts. First, the parasite infects a human (or any vertebrate host), and then it is transmitted from the infected human host (or infected vertebrate host) to the uninfected insect vector. The vectors of the *Plasmodium* species are mosquitoes of the genus Anopheles.^12^

**Figure 2:**
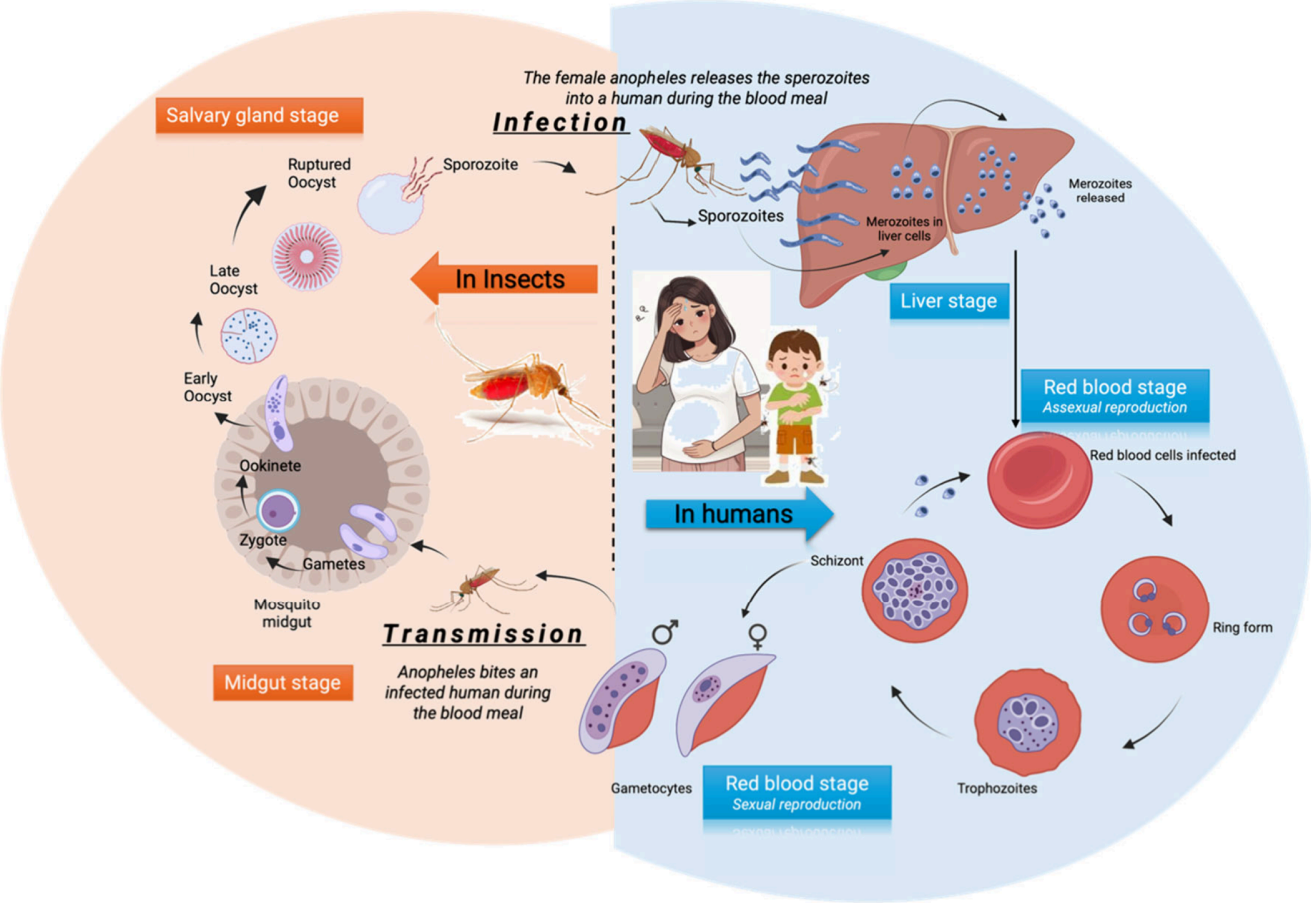
Life cycle of the malarial parasite.

The life cycle of malaria parasites (Figure 2) begins when parasites (sporozoites) produced in infected female *Anopheles* mosquitoes enter the blood of the vertebrate host after the bite. Sporozoites deposited in the dermis rapidly migrate to the liver and invade the hepatocytes, where they multiply by thousands through the process of schizogony. The resulting parasites, called merozoites, are released back into the blood and infect the erythrocytes. Inside an erythrocyte, a merozoite replicates asexually, and the parasites repeat this intraerythrocytic propagation cycle every 48 h for *P. falciparum*, *P. vivax*, and *P. ovale*, 24 h for *P. knowlesi*, and 72 h for *P. malariae*. Merozoites develop into a trophozoite ring, and a schizont forms. Mature schizonts are released after erythrocyte lysis, spreading the infection. The erythrocyte infection is established over the next 48 h. This blood cycle repeats itself, causing fever each time the parasites break free and invade new host cells. Some merozoites then differentiate into gametocytes. The sexual stage is faster in *P. vivax* than that in *P. falciparum*. This is because *P. vivax* continuously produces gametocytes, even in its early intraerythrocytic propagation cycles, whereas *P. falciparum* needs to complete several cycles of intraerythrocytic propagation before the differentiation into gametocytes begins.

The second part of the life cycle begins when the insect vector ingests blood containing gametocytes from an infected vertebrate host. The gametocytes are absorbed and mature in the mosquito’s digestive system, where they emerge as extracellular male and female gametes in the midgut. The gametes then fuse to form a zygote, which develops into an ookinete within 24 h. The ookinete migrates through the mosquito midgut epithelium and transforms into sporozoites that migrate to the salivary glands, ready to infect new hosts.^12,13^

**Figure 3:**
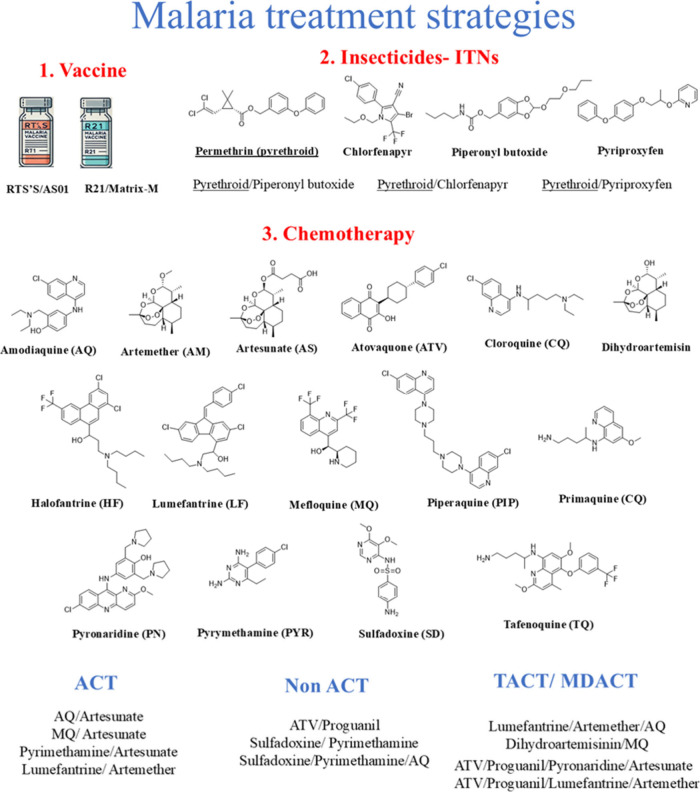
Strategies for the eradication of malaria: ACT: artemisinin-based combination treatments; TACT: triple artemisinin-based combination therapies; MDACT: multiple artemisinin-based combination therapies.

Several efforts have been made to resolve this major public health problem. The first was the development of an effective vaccine (Figure 3(1)). In fact, in October 2023, WHO recommended the use of safe and effective malaria vaccines RTS, SR21 (the first malaria vaccine), and R21 (the second vaccine) for the prevention of *P. falciparum* malaria in children living in malaria-endemic areas, with priority given to moderate and high transmission areas. The R21 or RTS vaccines are to be administered in a four-dose schedule starting at approximately 5 months of age^11^ These vaccines are promising advancements in the fight against malaria. However, substantial challenges and several obstacles remain.^14^ The second effort (Figure 3(2)) was to control the disease with insecticides, which play an important role in the prevention, control, and elimination of vector-borne diseases. Insecticide-treated nets (ITNs) have made a significant contribution to vector control, particularly in Africa. ITNs include pyrethroid (long-lasting insecticide), pyrethroid-piperonyl butoxide (PBO), pyrethroid-pyriproxyfen, and pyrethroid-chlorfenapyr. In many countries, ITNs are the only tool available for malaria vector control.^15^ This is an effective strategy, but the control of infected *Anopheles* mosquitoes with pyrethroid insecticides is becoming increasingly difficult due to the emergence of resistance to these insecticides. This is a growing problem threatening the effectiveness of vector control.^16^

The development of chemotherapeutics is the third longstanding effort (Figure 3(3)) to eradicate these deadly parasites. Inspired by the natural products, William Henry Perkin isolated the first antimalarial drug quinine and quinidine from the cinchona tree in 1856. The first synthetic drug, methylene blue, synthesized by P. Guttmann and P. Ehrlich, cured two malaria patients but was not used more widely. This set the stage for the development of other synthetic antimalarials. In the 1920s, researchers began to modify the structure of methylene blue and produced pamaquine in 1925, which still had several side effects. Primaquine (8-aminoquinoline class) introduced in 1952 was better tolerated. A major success was chloroquine (CQ), which became the mainstay of malaria treatment for more than two decades. From the mid-20th century, several quinoline derivatives have also been approved, such as amodiaquine (AQ) and primaquine (PQ), piperaquine, mefloquine (MQ), pyronaridine, and tafenoquine (TQ). The antimalarial drugs are classified by the stages of the malaria life cycle that they affect. Some act on the hepatic schizonts of the parasite stage and others on the asexual intraerythrocytic stages (blood schizonticidal drugs) of the parasites, while others can eliminate intraerythrocytic sexual (gametocytocidal) forms of the parasites and prevent transmission from human to mosquito.^17,18^

Nowadays, monotherapies are no longer used and have been replaced by combinations of two antimalarial drugs: proguanil and atovaquone (ATV), pyrimethamine and sulfadoxine, or artemisinin (artemether, artesunate, and dihydroartemisinin) with quinoline or nonquinoline derivatives such as lumefantrine, pyronaridine, or halofantrine. To counter the emergence of resistance to artemisinin-based combination treatments (ACTs), triple and multiple artemisinin-based combination therapies (TACT and MDACT) have been developed, which are in phase II/III clinical trials. Drug combination therapies have several limitations, including increasing parasite resistance against drugs, toxic side effects, and poor patient compliance.^18−21^ Given the impact of malaria, the development of well-designed metallodrugs to prevent and treat this deadly disease is essential.

As described in one of our previous reviews,^13^ the desired targets for the antimalarial drugs are as follows.

(a) Inhibition of heme aggregation to form hemozoin crystals, which is a major target of several antimalarial drugs such as chloroquine, amodiaquine, artemisine, etc.^19^ The heme moiety including Fe(II) is released in the digestive vacuole (DV) which can be rapidly oxidized to Fe(III) as ferriprotoporphyrin Fe(III)PPIX. This is considered toxic to the parasite. In the intraerythrocytic stage of *P. falciparum*, the degradation of hemoglobin to hemozoin within the acidic food vacuole is essential for the parasite’s survival in the host cell.^19^
(b) DNA of the parasite is another essential target. This has long been proposed as a potential target of the antimalarial activity of CQ. It was suggested to bind to DNA at relatively high drug concentrations via electrostatic forces, hydrogen bonds, van der Waals forces, and intercalation.^20,21^
(c) The cytochrome bc1 complex is a pivotal component of the respiratory chain. It targets the mitochondria of the parasite. One of the antimalarial drugs, atovaquone (ATV), degrades the mitochondrial membrane and interrupts the parasite’s energy supply.^22^
(d) The apicoplast is another parasite organelle, which, if inhibited, could lead to parasite death.
(e) The redox system of *P. falciparum* thioredoxin reductase (PfTrxR) and glutathione reductase (GR) is critical for the intraerythrocytic stages. It catalyzes the reduction of an important antioxidant protein, thioredoxin (Trx). Digestion of hemoglobin in infected erythrocytes results in the formation of superoxide anions, which must be reduced to H_2_O_2_ and subsequently converted to water by Trx. *P. falciparum* glutathione reductase (PfGR) catalyzes the reduction of glutathione disulfide (GSSG) to GSH. The tripeptide GSH is also essential for the parasite, as it detoxifies it from reactive oxygen species.^23^
(f) The cysteine protease falcipain-2 (FP-2), together with falcipain-3 and falcipain-2B, plays an essential role in the blood stage of *P. falciparum*, which validates it as target for new antimalarial drugs.

### 2.1 Zinc-Antimalarial Drug Complexes

Zinc-based complexes as antimalarial therapeutics are relatively unexplored and can offer big potential. Coordination of the antimalarial drug desferrioxamine (DFO) to the zinc ion yielded the Zn-DFO complex, which is thought to be more permeable into parasitized erythrocytes than the free DFO, and it was more effective against *Plasmodium falciparumin vitro*, particularly at concentrations below 20 μM. The Zn-DFO complex penetrates the cell and exchanges the zinc for ferric ion, rendering the iron unavailable for essential parasite functions.^24,25^

**Figure 4:**
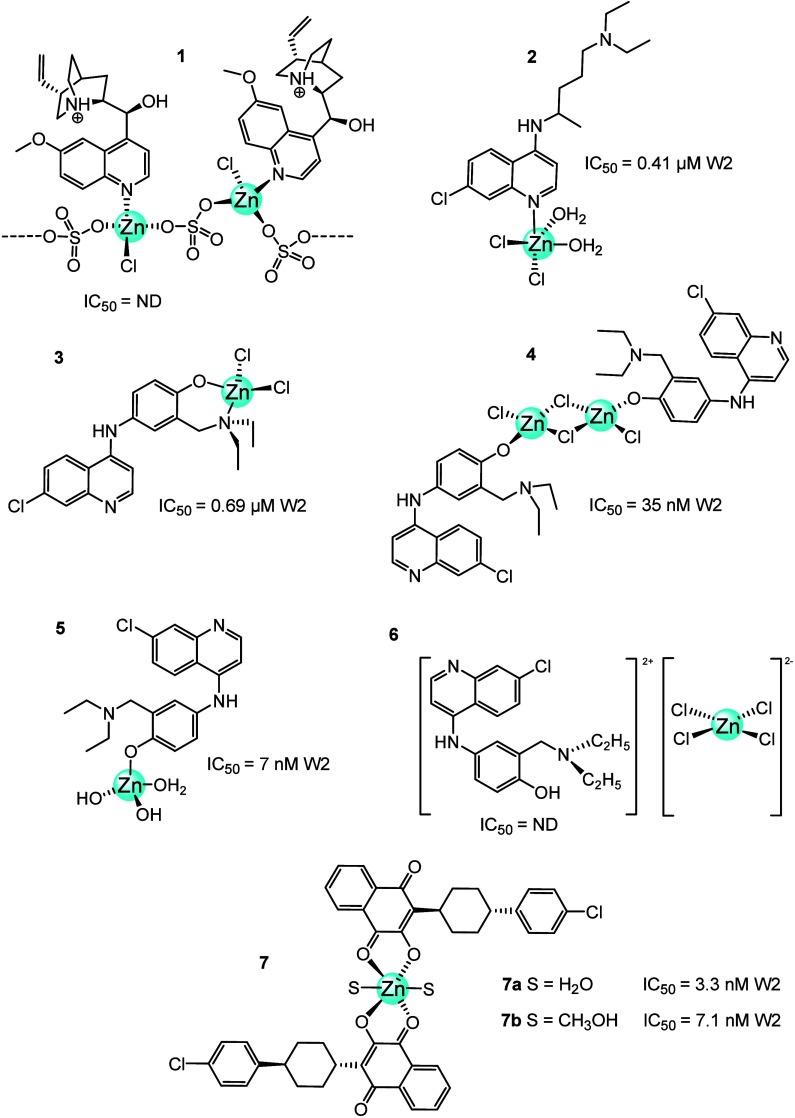
Zn-antimalarial drug complexes: W2: *Plasmosdium falciparum* chloroquine resistant strain; ND: not determined.

In 2009, quinine was coordinated to zinc to form a Zn-quinine complex (**1**, Figure 4), which was characterized by several techniques. Obaleye et al.^26^ determined its structure by X-ray diffraction analysis and described it as a tetrahedral geometry with coordinated oxygen, chlorine, and N(4) atom of the quinine hemisulfate in a polymer structure (chlorosulfato(2-ethenyl)-4-azabicyclo[2.2.2]oct-5-ylium(6-methoxyquinolin-4-yl)methanol zinc(II)). The antimalarial activity of this compound **1** against the growth of the *P. falciparum* parasite *in vitro* was three times more than that of quinine sulfate.^27,28^

Using the synergistic effect strategy, the Navarro research group coordinated the antimalarial drugs CQ, AQ, and ATV to zinc ion. These compounds were evaluated as inhibitors of cell proliferation of chloroquine-sensitive (CQ-S, 3D7) and chloroquine-resistant (CQ-R, W2) strains of *P. falciparum* parasite. Figure 4 shows the proposed structures of Zn-CQ complexes (**2** and **3**, Figure 4) and Zn-AQ complexes (**4** and **5**, Figure 4), which were tested against the (CQ-R, W2) strain. The results showed that compound **4** exhibited an IC_50_ of 7 nM, followed by compound **3** with an IC_50_ of 35 nM. Zn-AQ complexes were more active than Zn-CQ complexes. In compound **4,** zinc improved the potency of AQ by up to 12-fold. In addition, interaction studies with ferriprotoporphyrin have shown that the analyzed complexes exhibit similar interactions to those of the free drugs.^29^ A synthetic tetrachlorozincate salt of amodiaquine (**6**, Figure 4) was tested *in vivo* in male albino mice (*Mus musculus*), and the level of parasitemia was determined on day 4. The percentage reduction of parasitemia in groups treated with chloroquine and complex **6** showed no significant difference, and all animals in all test groups survived beyond the experimental period.^30^

Zn-ATV complexes **7a** and **7b** (Figure 4) showed promising antimalarial activity at nanomolar concentrations against *P. falciparum* strains, including those resistant to chloroquine (CQ-R W2 strains IC_50_ = 3.3 nM (**7a**) and 7.1 nM (**7b**)). They interacted not only with ferriprotoporphyrin but also with DNA, with a constant greater than 10^3^. This indicates a reversible interaction with this biomacromolecule. Viscosity studies suggest that these metal complexes interact with DNA by intercalative interaction, similar to chloroquine.^31,32^

### 2.2 Zinc-Diverse Ligand Complexes as Antimalarial Agents

**Figure 5:**
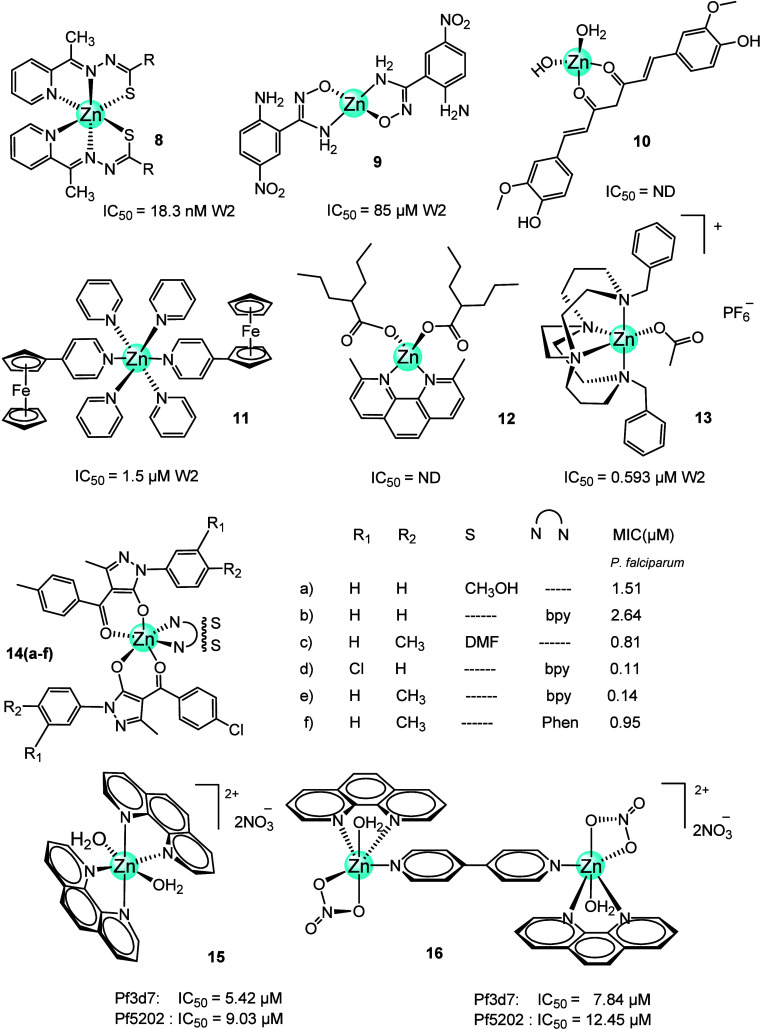
Zinc-diverse ligand complexes (**8**-**16**). W2: *P. falciparum* chloroquine-resistant; Pf3D7: drug-sensitive *Plasmodium falciparum*; 3D7 Pf5202: artemisinin-resistant *Plasmodium falciparum*.

In 2011, the synthesis of Zn(NNS)_2_ complex (**8**, Figure 5) and its biological evaluation against the malaria parasite *P. falciparum* were reported.^33^ NNS is the tridentate dithioester 3-[1-(2-pyridyl)ethylidene]hydrazinocarbodithioate. This octahedral Zn(NNS)_2_ complex exhibited significant potency against both cysteine protease enzymes falcipain-2 (FP-2, 13.850 nM) and falcipain-3 (FP-3, 8.462 nM), and against the chloroquine resistant W2 strain (18.3 nM) with an exceptional selectivity (*S* = 135.2), while the free NNS ligand displayed moderate activity against the both FP-2 and FP-3 and good activity against W2.^33^

Lautre et al.^34^ designed inhibitors of purine nucleoside phosphorylase (PNP) in *P. falciparum* (PfPNP), which is an essential enzyme for the growth and survival of the malaria parasite. The malfunction of this enzyme in *P. falciparum* disrupts the catalysis of the phosphorolysis of inosine to ribose-1-phosphate and the production of hypoxanthine required for sustained growth. The Zn(II)-ANHB complex (**9**, Figure 5) (ANHB: 2-amino-5-nitro-*N*-hydroxybenzamidine) and other metal derivatives, such as Co(II)-ANHB and Cr(II)-ANHB, were prepared and tested for inhibition of both the growth of parasite *P. falciparum* and PfPNP. The *in vitro* antiplasmodium activity of the ligand and the Zn derivative were EC_50_ (half-maximal effective concentration) = 115 μM and EC_50_ = 85 μM, respectively. In comparison, the chloroquine had EC_50_ = 53 μM. The *K*_i_ values of Zn(II)-ANHB complex **9** and the free ANHB ligand against PfPNP were 35 and 85 μM, respectively. These results clearly indicated that complex **9** is a better PfPNP inhibitor than the free ligand.^34^

Laware and Kuchekar^35^ prepared the Zn-curcumin complex (**10**, Figure 5) by mixing zinc sulfate and curcumin, a natural polyphenolic compound with antimalarial activity. Zn improved the solubility, stability, and bioavailability of curcumin. Mouse survival and % parasitemia were studied in *P. berghei*-infected mice treated with 5 mg/day of curcumin, artemether, complex **10**, and a combination of **10** with artemether. The parasitemia was measured on day 4. Complex **10** reduced it by 30.9%, showing superior antimalarial activity to curcumin (reduced parasitemia by 4.22%). The combination of complex **10** and artemether increased survival to 100% and reduced parasitemia even further (90.86%). This suppressive effect was superior to the drug alone at the same dose.^35^

Azam et al. used a ferrocenyl terpyridine derivative (Fctpy). It is well-known that terpyridines are tridentate ligands, and structural changes are limited when the oxidation state of the center changes. Metal complexes of terpyridines are generally known as DNA binders^36,37^ and described as antiproliferative agents. This makes them attractive chemotherapeutic agents for cancer and infectious diseases.^38−40^ The [Zn(Fctpy)_2_](PF_6_)_2_ complex (**11**, Figure 5) exhibited an IC_50_ value of 1.5 μM as an inhibitor of *P. falciparum* versus 3.0 μM for the free Fctpy. **11** is more potent than the ligand (Fctpy) alone, suggesting that the presence of a coordinated metal around Fctpy is essential for antimalarial activity enhancement. Similarly, the low potency of Fctpy in the inhibition of the hemin polymerization was improved in its metal derivatives, to the level comparable to that of chloroquine. These results suggested that the antimalarial activity of these compounds is attributable to the inhibition of the hemein polymerization in the parasite cells.^41^ The authors believed that the metal complexes dissociated in the cellular media, releasing the metal ion, which interacted within the parasite cell, and caused toxic effects to the parasite.^42,43^

Ali and co-workers prepared a series of zinc complexes using another bioactive ligand, sodium valproate. Valproate is an anticonvulsant drug that has also been shown to be effective against other diseases such as bipolar disorder, migraine, and neuropathic pain.^44^ The precursor Zn-valproate complex was reacted with each of the following bioactive nitrogen base compounds: 2,9-dimethyl-1,10-phenanthroline (2,9-dmphen), quinoline (quin), 2-aminopyridine (2-ampy), and 2-amino-6- picoline (2-ampic). The antiplasmodial activity of the [Zn(valproate)(nitrogen base)] complexes was determined through the inhibitory effect on β-hematin formation, compared to antimalarial drugs chloroquine and amodiaquine with a similar mechanism of action. The antimalarial efficiency was reported as a percentage yield of β-hematin formation. The lower the yield, the more efficacious the drug is considered. In the presence of complex **12** (Figure 5) this was 20%, and AQ and CQ resulted in 9%, both at a concentration of 0.4 mM compared to the negative control (DMSO) at 83%. Other [Zn(valproate)(nitrogen base)] complexes did not show any significant inhibitory effect.^45^

Hubin et al.^46^ reported a series of M-bridged tetraazamacrocyclic chelators, where M is a divalent metal such as manganese(II), iron(II), cobalt(II), nickel(II), copper(II), and zinc(II). All compounds were tested *in vitro* against *P. falciparum* chloroquine-resistant (W2) and chloroquine-sensitive (D6) strains. [Zn(cyclam analogue)(OAc)]PF_6_ complex (**13**, Figure 5) showed better activity against both strains than the free cyclam. For instance, on the D6 strains the cyclam analog ligand had IC_50_ = 2.679 μM, and the complex **13** showed IC_50_ = 0.342 μM. The corresponding values against W2 strains were 4.358 and 0.593 μM for the ligand and **13**, respectively. The manganese complexes were even more effective (IC_50_ = 0.157 μM (D6) and 0.127 μM (W2)). However, none reached the level of the positive controls, chloroquine (IC_50_ = 0.037 μM (D6) and 0.679 μM (W2)) and artemisinin (IC_50_ = 0.024 μM (D6) and 0.004 μM (W2)).^46^

In 2019, Jadeja and co-workers^47^ reported the synthesis of 4-acyl pyrazolone derivatives (PCBPMP: 4-(4-chlorobenzoyl)-5-methyl-2-phenyl-2,4-dihydro-3*H*-pyrazol-3-one; PCBTPMP: 4-(4-chlorobenzoyl)-5-methyl-2-(*p*-tolyl)-2,4-dihydro-3H-pyrazol-3-one) and its Zn(II) complexes [Zn(PCBPMP)_2_(CH_3_OH)_2_] (**14a**, Figure 5), [Zn(PCBPMP)_2_(bpy)] (**14b**, Figure 5), and [Zn(PCBTPMP)_2_(DMF)_2_] (**14c**, Figure 5). The antimalarial activity of these compounds was evaluated against *P. falciparum*. The Zn complexes (**14a**, MIC = 1.51 μM; **14b**, MIC = 2.64 μM; **14c**, MIC = 0.81 μM) were more active than the free ligands (PCBPMP, MIC = 4.51 μM; PCBTPMP, MIC = 2.97 μM), but less active than chloroquine. Complex **14c** was also the leading compound in a molecular docking study for falcipain 2, plasmepsin 1, and M1 aminopeptidase.^47^ A year later, the same research group reported similar work with the acyl pyrazolones as the main ligand and used 2,2̀-bipyridine (bipy) or 1,10-phenanthroline (phen) as auxiliary ligands to complete the distorted octahedral coordination sphere of the Zn ion. The compounds and their activities were as follows: [Zn(PClBPMP)_2_(bpy)] (**14d**, PClBPMP = 4-(4-chlorobenzoyl)-2-(3-chlorophenyl)-5-methyl-2,4-dihydro-3*H*-pyrazol-3-one), MIC = 0.11 μM; [Zn(PCBTPMP)_2_(bpy)] (**14e**, Figure 5), MIC = 0.14 μM; [Zn(PCBTPMP)_2_(phen)] (**14f,**Figure 5), MIC = 0.95 μM. The acyl pyrazolone ligands and the three Zn derivatives exhibited a significant level of inhibition of *P. falciparum* growth. Every Zn complex was more active against *P. falciparum* than the free ligand and the previously reported compounds, complex **14d** (Figure 5) being the most active.^48^

Recently, Lai and co-workers^49^ reported mononuclear and dinuclear Zn(II) and Cu(II) complexes using phen and 4,4′-bipyridine (4,4’bipy) as a bridging ligand for the dinuclear metal complexes. The zinc(II) complexes **15** and **16** (Figure 5) were effective against the drug-sensitive Pf3D7 strain with an excellent therapeutic index but encountered total resistance in the drug-resistant Pf5202 strain, unlike the copper(II) complexes, which were potent against both strains. Similarly, only the copper(II) analogs induced increased hemolysis of the red blood cells (RBCs) with increasing concentration. Investigations into the possible mechanisms of action focused on the induction of ROS, inhibition of the malarial proteasome, loss of mitochondrial membrane potential, and morphological features indicative of apoptosis. The zinc(II) complexes **15** and **16** (Figure 5) did not generate any ROS as the fold increase in ROS was negative and they were poor inhibitors of the three proteolytic sites of the 20S proteasome of the Pf3D7 and Pf5202 lysates (20S proteasome at chymotrypsin-like (CT-L) site, trypsin-like (T-L) site, and caspase-like (C-L) site proteolytic sites).^49^

Interestingly, zinc supplementation has long been studied as a potential intervention to reduce the risk of malaria infection. However, there is conflicting evidence that either alone or in combination with other micronutrients such as vitamin A, iron, or others, zinc does not significantly alter the risk of malaria parasitemia.^50^ This opens the door to the belief that the way to combat this deadliest disease is through the use of coordination compounds that contain zinc and the appropriate ligands in one molecule, acting as a multitarget drug.

## 3 Zinc Complexes against *Leishmania* Parasites

Leishmaniasis is a group of diseases caused by intracellular protozoa of the genus *Leishmania*, belonging to the family Trypanosomatidae and the order Kinetoplastidae. The parasites are transmitted by about 30 species of phlebotomine sandflies. The World Health Organization (WHO) reports that leishmaniasis remains a major public health challenge affecting millions of people worldwide. Leishmaniasis is endemic in 99 countries, with an estimated 700,000 to 1 million new cases each year. There are three main forms of leishmaniasis: visceral (VL), cutaneous (CL), and mucocutaneous (ML). VL is the most severe form and is almost always fatal if untreated, while CL is the most common and typically results in skin ulcers.^51^

**Figure 6:**
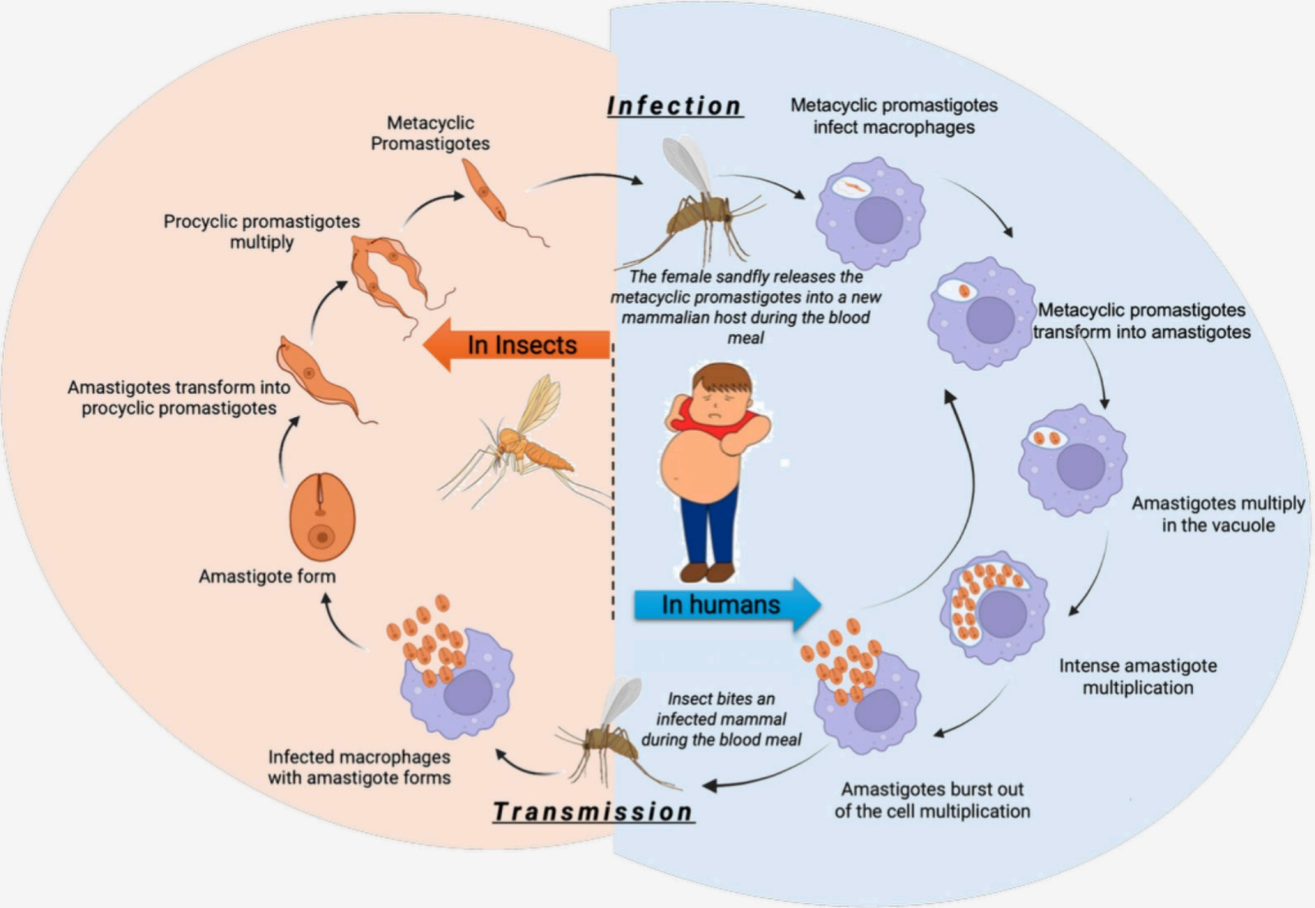
Life cycle of the *Leishmania* parasite.

The *Leishmania* parasite life cycle alternates between two hosts: mammals and insect vectors (Figure 6). It has two distinct stages: promastigotes (motile stage), which reside in the intestines of the sandflies, and amastigotes (nonmotile), which are found in the macrophages of the vertebrate host (humans or animals). The cycle starts when a female sandfly releases these metacyclic promastigotes into a new mammalian host through regurgitation. Once inside the new host, metacyclic promastigotes can infect macrophages. Within the macrophages, these metacyclic promastigotes transform into amastigotes. Amastigotes attach to the membrane of the parasitophorous vacuole and begin to multiply within this vacuole. This multiplication becomes intense, leading to bursting of the host cell. The released amastigotes then infect new macrophages. In the transmission, the sandfly bites an infected mammal and sucks the blood of the infected macrophages with the amastigotes. These amastigotes then transform into procyclic promastigotes. Procyclic promastigotes multiply within the midgut of the sandfly. These promastigotes migrate toward the stomodeal valve in the anterior midgut and resume cell division. Subsequently, promastigotes transform into infective metacyclic promastigotes (WHO, 2023).^51^

A vaccine against leishmaniasis is not available. Insecticides are used as a control strategy, known as indoor residual spraying (IRS). The use of DDT was completely stopped after 2016, and only pyrethroids are used overall. The effectiveness of the insecticide use for leishmaniasis control is lower than for malaria.^15^

Current chemotherapy has many disadvantages, and the development of new, effective treatments is urgent. Combination therapies for cutaneous and visceral leishmaniasis are being increasingly studied, and repurposing drugs such as artesunate, used in the treatment of malaria, can also be a promising approach to combat the disease.^52^

**Figure 7:**
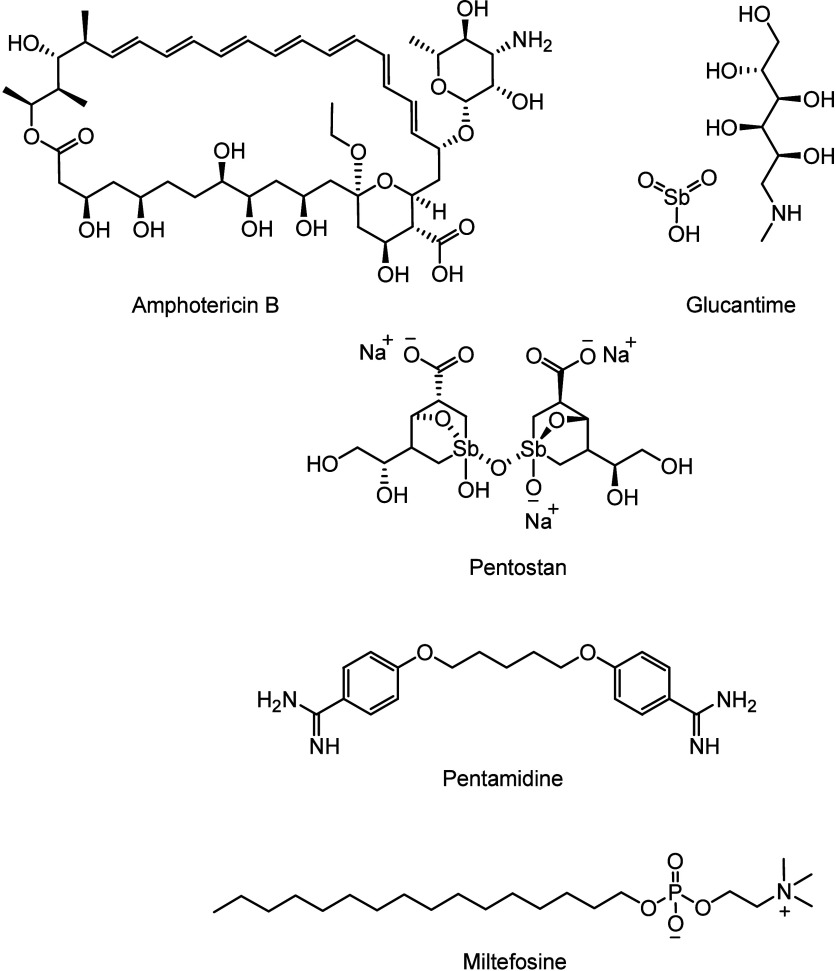
Chemotherapies against leishmaniasis.

Figure 7 shows the limited treatment available for various forms of leishmaniasis, based on pentavalent antimonial compounds such as sodium stibogluconate (Pentostam) and meglumine antimoniate (Glucantime). Their efficacy varies among endemic regions and *Leishmania* species, and treatment resistance is also an increasing problem.^53^

Figure 7 also shows alternative drugs to treat this disease. Amphotericin B is used especially in areas with high resistance to antimonials, with its liposomal formulation being preferred due to lower toxicity. However, this formulation has high costs, which hinder the broad use of this medication. Pentamidine is another option for resistant strains, although it is associated with more side effects. Miltefosine, an oral treatment, has shown efficacy and is particularly useful in regions with a limited healthcare infrastructure. In India, resistance to antimonials is high, leading to the increased use of liposomal amphotericin B. In Latin America, Glucantime is still widely used, but resistance is a growing concern. Miltefosine is becoming an important alternative to pentavalent antimonials. As the choice of treatment depends on region, species of *Leishmania*, and individual response, continuous monitoring of drug resistance is essential.^54^

Understanding the biology of *Leishmania* and the host interaction is essential for the development of new treatments.^55−57^ Omics has contributed greatly to this by identifying new targets for leishmaniasis treatment. *Leishmania* is not actively targeted by immune cells, so the parasites can infect macrophages and dendritic cells through cell surface receptors, ensuring their survival and proliferation in the host. This specific interaction is critical for maintaining infection, and it represents a potential target for intervention. Zinc complexes represent a promising approach to the leishmaniasis treatment.

In 2014, Bafghi et al. reported the antileishmanial activity of ZnSO_4_·7H_2_O (zinc sulfate heptahydrate). The results were promising, the LD_50_ (median lethal dose) of the Zn(II) salt were 44.4 and 32.4 mg/mL, significantly lower than those of Glucantime (LD_50_ = 334.7 mg/mL, 2250 mg/mL) against *L. major* and *L. tropica*, respectively. This suggests that zinc derivatives may be a safe and effective alternative for the treatment of cutaneous leishmaniasis.^57,58^

**Figure 8:**
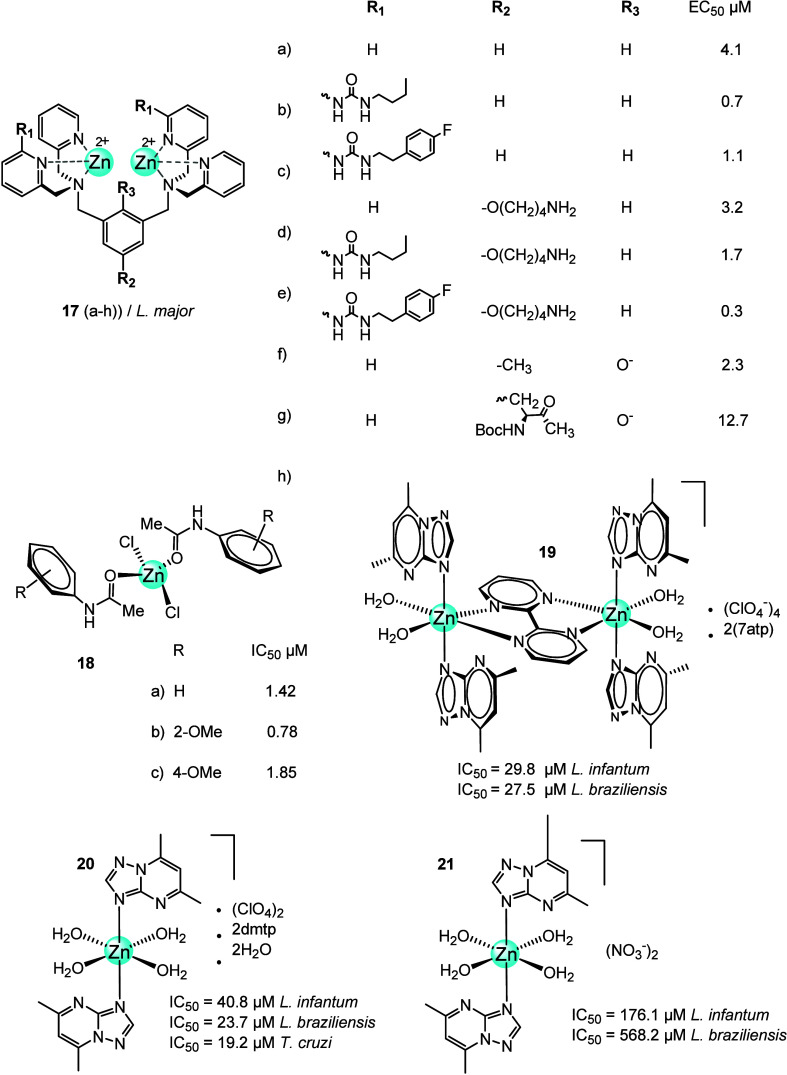
Chemical structures of aryl acetamides and triazolopyrimidine Zn complexes (**18**–**21**).

Rice et al. described eight different zinc(II)-dipicolylamine complexes (**17a**–**h**, Figure 8) and their activity toward *L. major*, one of the species responsible for cutaneous leishmaniasis.^59^ The *in vitro* biological assays with complexes **17a**–**h** showed moderate to strong activity, with minimal cytotoxicity to mammalian cells. The zinc complexes showed selective toxicity against the *L. major* promastigote forms with EC_50_ in the range of 0.3–12.7 μM. Although the most active compound was **17g**, **17h** was selected for *in vivo* studies in the mouse footpad infection model due to its structural simplicity and ease of preparation. Five weekly intralesional doses of **17h** resulted in an ∼70% reduction in parasite burden compared to an untreated cohort. The dose of **17h** was approximately 50 times lower than the positive control (antimonial), yet the reduction in infection burden over 12 days was very similar. In addition, treatment with **17h** resulted in significantly less host tissue damage at the treatment site compared with the control antimonial treatment. *In vitro* activity and toxicity measurements showed that **17h** is quite active against intracellular amastigotes and is much less toxic to murine macrophages. The mechanism of action of **17h** against *L. major* is not clear and may be multifactorial. The author suggested that these Zn complexes may disrupt the parasite’s cell membrane and alternatively alter the metal cation concentrations within the cytosol.

Iqbal and co-workers^60^ prepared three Zn(II) complexes (**18a**–**c**) (Figure 8) of different aryl acetamides and tested them for their inhibition of carbonic anhydrase (CA) and alkaline phosphatase. Complexes **18a**–**c** proved to be better inhibitors of CA (IC_50_ 0.91, 0.81, and 4.72 μM) than the corresponding ligands (IC_50_ 7.57–4.21 μM), and were comparable to acetazolamide (standard inhibitor). Similar, excellent behaviors were observed in alkaline phosphatase inhibition. The aryl acetamide ligands (**a-c**) and their zinc derivatives (**18a**–**c**) were tested *in vitro* against the promastigote forms of *Leishmania major*. The IC_50_ values of the ligands **a**–**c** were 5.12, 3.05, and 4.03 μM, respectively, and those of the Zn complexes **18a**–**c** were 1.42, 0.78, and 1.85 μM, respectively. Complex **18b**, containing an ortho-methoxy substituent on the aryl ring, was the most active. However, its activity was less than that of the antileishmanial amphotericin B (IC_50_ 0.29 μM).^60^

Triazolopyrimidine derivatives are considered analogs of the nucleobase hypoxanthine and have been the focus of several biochemical studies due to their diversified pharmacological activities.^61^ A series of transition metal-based triazolopyrimidine complexes have been prepared and evaluated against cancer and parasitic diseases.^62−64^ In this section, we focus on the zinc complexes of triazolopyrimidine derivatives, such as 7-amino-1,2,4-triazolo[1,5-*a*]pyrimidine (**7atp**), 5,7-dimethyl-1,2,4-triazolo[1,5-*a*]pyrimidine (**dmtp**), 2,4-triazolo[1,5-*a*]pyrimidine (**tp**), and 7-oxo-5-phenyl-1,2,4-triazolo[1,5-*a*]pyrimidine (**ftpO**), and their activity against *Leishmania* parasites.^65,66^

In 2012, a [Zn_2_(7atp)_4_(μbpym)(H_2_O)_4_](ClO_4_)_4_·2(7atp) complex (**19**, Figure 8), and four other metal complexes of triazolopyrimidine derivatives (tp, dmtp, and 7atp) were synthesized and structurally characterized. The coordination sphere of complex **19** contains 7atp and the chelating bridging ligand bipy. The triazolopyrimidines coordinated monodentately via N3 in all studied metal complexes as confirmed by X-ray. The therapeutic potential of the compounds was evaluated *in vitro* against *Leishmania infantum* and *Leishmania braziliensis*. The free 7atp (IC_50_*L. infantum* 56.4 μM, *L. braziliensis* 61.8 μM) was less active than **19** (IC_50_*L. infantum* 29.8 μM, *L. brasiliensis* 27.5 μM). **19** was much less cytotoxic toward macrophages and more selective against parasites. However, both compounds exhibited lower antiparasitic activities than the reference drug Glucantime (IC_50_*L. infantum* 18.0 μM, *L. brasiliensis* 25.6 μM).^67^

In 2014, metal complexes of dmtp such as [M(dmtp)_2_(H_2_O)_4_](ClO_4_)_2_·2dmtp·2H_2_O (M = Mn, Fe, Co, Ni, Zn) and [Cu(dmtp)_4_(H_2_O)_2_](ClO_4_)_2_·2H_2_O were described.^68^ These metal complexes were characterized by using X-ray, spectroscopic, and thermal methods. Both *in vitro* and *in vivo* (murine model) studies were conducted to evaluate their antiproliferative activity against *Leishmania* spp. [Zn(dmtp)_2_(H_2_O)_4_](ClO_4_)_2_·2dmtp·2H_2_O complex (**20**, Figure 8) exhibited significant activity against promastigote forms (IC_50_*L. infantum* 42.4 μM, *L. braziliensis* 65.4 μM) and also amastigote forms (IC_50_*L. infantum* 40.8 μM, *L. braziliensis* 23.7 μM). However, its activity was slightly lower than that of Glucantime.^68^

Six years later, a series of transition metal-dmtp complexes were prepared by reacting dmtp with M(NO_3_)_2_·*x*H_2_O (M = Cu(II), Co(II), Ni(II) and Zn(II)). [Zn(dmtp)_2_(H_2_O)_4_](NO_3_)_2_ (**21**, Figure 8) had the same coordination sphere as **20** but was less active in *in vitro* studies against promastigote forms of the same *Leishmania* spp. (*L. infantum* and *L. braziliensis*) than **20**. Complex **21,** like **19** and **20**, was less cytotoxic and more selective for the parasite than Glucatime and free dmtp (J774.2 macrophages: **21** = 542.5 μM, Glucantime = 15.2 μM, and dmtp= 98.7 μM) (IC_50_ 176.1 μM in *L. infantum* and 568.2 μM in *L. braziliensis*).^69^ Recently, enzymatic assays with first-row transition metal-dmtp complexes were reported to elucidate the mechanisms of action. The metal complexes showed high antiparasitic efficacy with superoxide dismutase enzymatic assays indicating distinct behaviors depending on the thermochromic form tested. Both the antiproliferative and enzymatic assays confirmed synergistic leishmanicidal activity of these metal complexes, with coordination sphere changes due to thermochromism affecting their physical properties and biological efficacy.^70^

**Figure 9:**
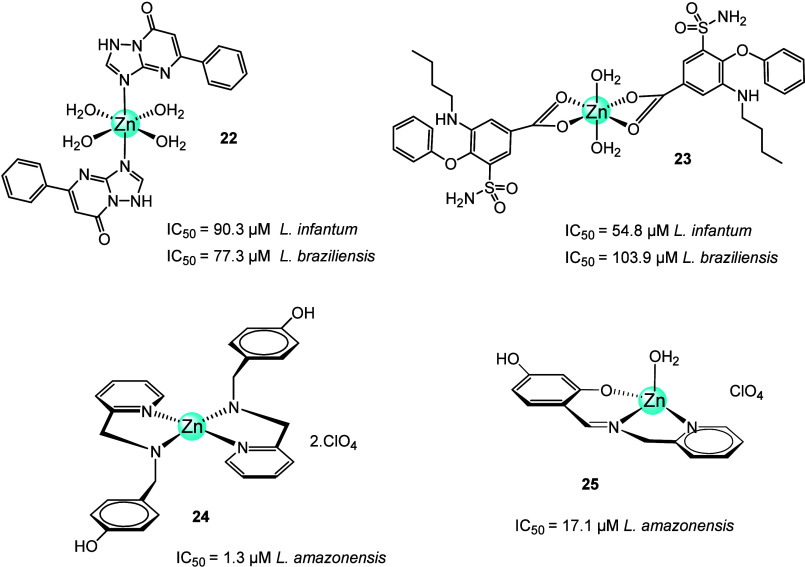
Chemical structures of Zn complexes of triazolopyrimidine, bumetanide, or aminopyrydyl ligands (**22**–**25**).

In 2023, metal complexes of ftpO [M(ftpO)_2_(H_2_O)_4_] (M = Cu, Co, Ni, Zn) were prepared and tested *in vitro* against five different species of *Leishmania* spp. (*L. infantum*, *L. braziliensis*, *L. donovani*, *L. peruviana*, and *L. mexicana*). The [Zn(ftpO)_2_(H_2_O)_4_]complex (**22**) (Figure 9) showed moderate or significant effect against the promastigote forms of *Leishmania* spp. (IC_50_*L. infantum* = 90.3 μM, *L. braziliensis* = 77.3 μM, *L. peruviana* >200 μM, *L. mexicana* >200 μM, *L. donovani* = 51.3 μM), but the IC_50_ was higher than that of the antimonial drug. Although it did not exceed the performance of the control drug, it was less cytotoxic.^71^ Among complexes with other metals, the zinc analogs were less active but showed an improved selectivity index (SI). The best of these Zn complexes (**19**–**22**) was [Zn(dmtp)_2_(H_2_O)_4_](NO_3_)_2_ (**21**) .

Because of promising results with the metal complexes of various triazolopyrimidine ligands, the same research group took a step further and used a therapeutic ligand, bumetanide (Hbum), to synthesize a zinc-bum complex and investigate its leishmanial activity against three species of *Leishmania* (*L. infantum*, *L. braziliensis*, and *L. donovani*). [Zn(bum)_2_(H_2_O)_2_]·2H_2_O complex (**23**, Figure 9) showed antiparasitic activity against all three species (IC_50_*L. infantum* = 54.8 μM, *L. braziliensis* = 103.9 μM, *L. donovani* = 45.1 μM). However, the IC_50_ values were significantly higher than those of Glucantime. Nevertheless, both compounds had high cytotoxicity levels (over 1000 μM), which improved the SI for *L. infantum* and *L. donovani* more than 20 times. The synergistic effect, by the coordination to Zn(II) in complex **23,** enhanced the antiparasitic activities of bumetanide (IC_50_ = 196.5 μM) against *L. braziliensis*.^72^

In 2017, Velasco-Torrijos and co-workers^73^ reported the synthesis and anti-*Leishmania amazonensis* activity of two compounds, bis(*N*-[4-(hydroxyphenyl)methyl]-2-pyridinemethamino)zinc perchlorate monohydrate (**24**, Figure 9) and (5-hydroxy-2-{[(2-methylpyridinyl)-*E*-imino]methyl}phenolate) zinc perchlorate monohydrate (**25**, Figure 9). The compound **24** had the highest leishmanicidal activity (IC_50_ = 1.3 μM) compared to **25** (IC_50_ = 17.1 μM) and the ligand (IC_50_ = 25.3 μM), a significant increase in the activity of the ligand. **24** was also less toxic than the free ligand and amphotericin B as a reference drug. However, it was less active than amphotericin B (IC_50_ = 0.012 μM).^73^

The morphological and physiological alterations induced by **24** on *Leishmania amazonensis* promastigotes and its effect against the intramacrophage amastigotes (IC_50_ = 3.91 μM) were studied by the same research group in 2021. Complex **24** promoted a progressive reduction in the promastigote size and a remarkable increase in granularity and complexity as assessed by flow cytometry. Transmission electron microscopy (TEM) analysis revealed extensive mitochondrial and plasma membrane changes, although plasma membrane integrity was not compromised. A decrease in mitochondrial dehydrogenase activity was also observed with increased production of reactive oxygen species. Promastigote form also showed changes in lipid metabolism.^74^

**Figure 10:**
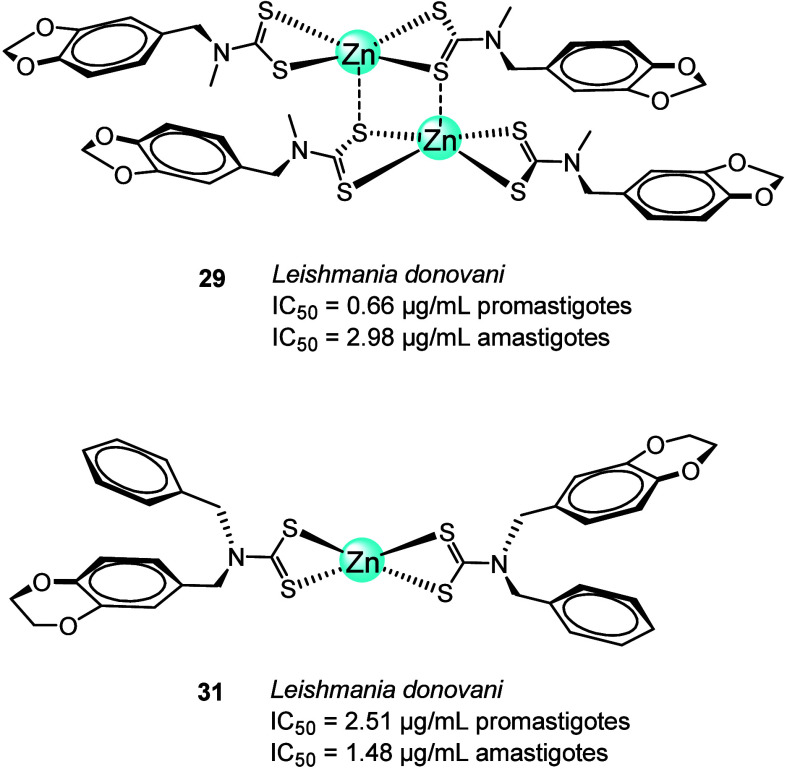
Chemical structures of Zn complexes **29** and **31** containing dithiocarbamate ligands.

Singh and co-workers^75^ reported the synthesis and characterization of six homoleptic zinc(II) dithiocarbamate complexes (Figure 10) accordingly with L = *N*-ferrocenyl-*N*-methyldithiocarbamate (**26**), *N*-benzo[*d*][1,3]dioxol-5-ylmethyl)-*N*-furfuryl dithiocarbamate (**27**), *N*-benzo[*d*][1,3]dioxol-5-ylmethyl)-N-benzyl dithiocarbamate (**28**), *N*-benzo[*d*][1,3]dioxol-5-ylmethyl)-*N*-methyl dithiocarbamate (**29**), *N*-ethylmorpholine-*N*-(4-methoxyphenylmethyl) dithiocarbamate (**30**), and *N*-(benzo[*d*][1,4]dioxol-6-ylmethyl)-*N*-benzylmethyl dithiocarbamate (**31**). The six complexes were tested *in vitro* against the promastigote forms and intracellular amastigotes using miltefosine as a reference. Compounds **29** and **31** were the most active in promastigote forms (IC_50_ 0.66 μg/mL (**29**) and 2.51 μg/mL (**31**)) and in amastigote forms (IC_50_ 2.98 μg/mL (**29**) and 1.48 μg/mL (**31**)) of *L. donovani*. Their cytotoxicity was also low. Electron microscopy analysis of promastigote forms revealed loss of flagellum and rounded morphology with a substantial reduction in size compared to the control promastigotes. Cell shrinkage and cell condensation were also observed as evidence of apoptosis. *In vivo*, these compounds showed 80% parasitic inhibition at 50 μg/mL. Their promising activities can be attributed to the increased electron density on the N atom of the dithiocarbamate unit, due to the +I effects of the methyl group in **29** and the substituted benzene ring in **31**.^75^

**Figure 11:**
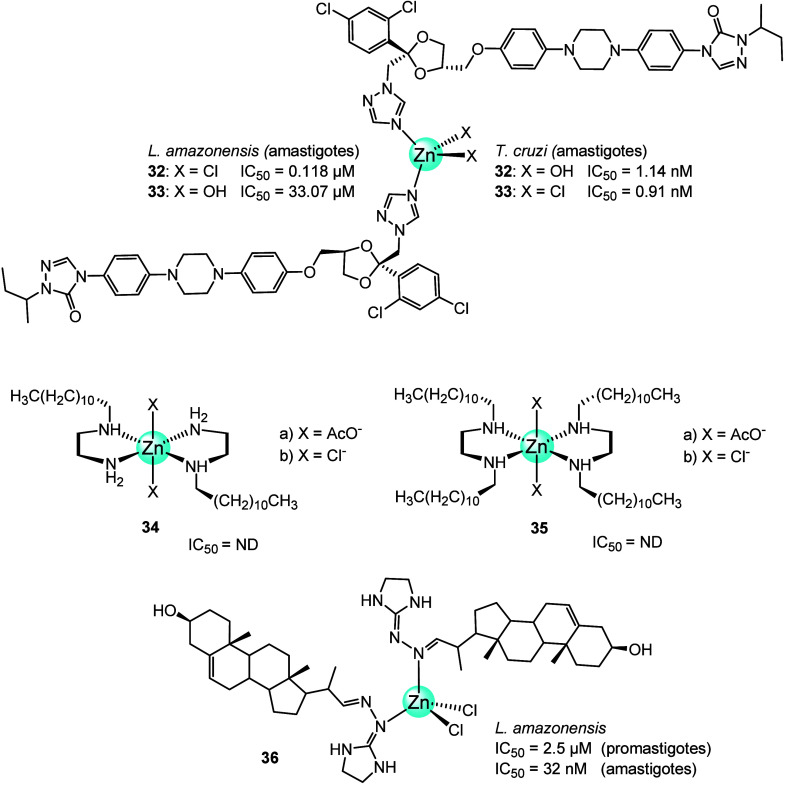
Chemical structure of Zn complexes of itraconazole, diamine, and azasterol (**32**–**36**).

In 2020, Navarro and co-workers^76^ synthesized two Zn-ITZ complexes, [Zn(ITZ)_2_Cl_2_] (**32**; Figure 11) and [Zn(ITZ)_2_(OH)_2_] (**33**; Figure 11), where itraconazole (ITZ) is a well-known antifungal agent, and evaluated their biological activities against protozoan parasites (*L. amazonensis*, *T. cruzi*, and *T. gondii*) and fungi.^76^ The antiproliferative effects of the zinc complexes and ITZ against the intracellular amastigotes of *L. amazonensis* (the clinically relevant stage of this parasite) showed IC_50_ values of 145.38, 0.118, and 33.07 nM for ITZ, **32**, and **33**, respectively. Both Zn-ITZ complexes were much more potent than the ligand alone (ITZ), but complex **32** in particular decreased the effective concentration of itraconazole 1200-fold. Both zinc complexes also exhibited remarkably high SI, **32** with the biggest increase compared to ITZ (SI = 5084 vs 103.17).

Scanning electron microscopy (SEM) and transmission electron microscopy (TEM) analyses of *L. amazonensis* promastigotes revealed several morphological changes in the parasite after treatment with ITZ, **32**, and **33**. Promastigotes appeared to be rounded and swollen, sometimes presenting more than two flagella in the SEM images. Several ultrastructural changes were also observed by TEM. These included intense mitochondrial swelling, followed by disorganization, the presence of lipid bodies, and the presence of vacuoles similar to autophagosomes, some of which contained small vesicles and membrane profiles. Additionally, morphological and ultrastructural analyses revealed several changes in the shape of the cell body, membrane structure, and presence of lipid bodies, which could indicate a possible inhibition of ergosterol biosynthesis. Also, the presence of large vacuoles similar to autophagosomes could be related to autophagy and increased activity in the remodeling of abnormal lipids and organelles affected by the treatment. It is assumed that the changes in chromatin condensation could be induced by the zinc ion.^76^

It is important to emphasize that complexes **32** and **33** showed a broad spectrum of antiparasitic and antifungal activities at lower concentrations than the ITZ in each microorganism studied. The strategy of combining zinc with ITZ proved to be efficient in enhancing ITZ activity and reducing toxicity. Interestingly, Zn(ITZ)_2_Cl_2_ (**32**) performed better against *L. amazonensis*, *T. gondii*, *S. brasiliensis*, and *S. schenckii* than did Zn(ITZ)_2_(OH)_2_ (**33**). This opens further perspectives for future applications of these Zn-ITZ complexes in the treatment of parasitic disease and sporotrichosis.

In 2022, Navarro and co-workers^77^ synthesized a series of Zn-diamine complexes (Figure 11) of *N*-alkylated (*N*-dodecyl-1,2-ethylenediamine (**L1**)) and *N*,*N*′-dialkylated (*N*,*N*′-didodecil-1,2-ethylenediamine (**L2**)). The zinc complexes were [Zn(**L1**)_2_(AcO)_2_] (**34a**), [Zn(**L1**)_2_Cl_2_] (**34b**), [Zn(**L2**)_2_(AcO)_2_] (**35a**), and [Zn(**L2**)_2_Cl_2_] (**35b**). The antiparasitic activity of these compounds was tested on *L. amazonensis* promastigotes. All Zn(II) complexes had higher biological activity than the corresponding free ligand (**L1** or **L2**), with **34b** and **35b** being the highest, IC_50_ 1 μM to 5 μM.

The morphology and ultrastructure of *L. amazonensis* promastigotes after treatment with free ligands and the Zn derivatives were investigated by SEM and TEM. According to the SEM image of the *Leishmania* cell body after treatment with **35b,** intense remodeling of the morphology of the *Leishmania* parasite was induced, such as rounding and reduction of its cell body and roughness of the cell surface in comparison with the control cells. Also, more than one flagellum was visible. This could indicate a possible interruption in the cell cycle, potentially leading to the parasite’s death. The ultrastructural effects in *L. amazonensis* promastigotes induced by complex **35b** were determined by TEM. Several changes were noticed, like abnormal chromatin condensation, swelling in the kinetoplast region, and swelling in the medial region of the parasite cell body, while the posterior region became thinner. In addition, the presence of myelin-like and autophagic vacuoles, nucleus, mitochondrion, kinetoplast, and lipid bodies were also observed.^77^

Recently, Visbal et al.^78^ reported the synthesis, characterization, and biological evaluation against *L. amazonensis* of the ZnCl_2_(**H3**)_2_ complex (**36**, Figure 11). The ligand **H3** (22-hydrazone-imidazoline-2-yl-chol-5-ene-3β-ol) is a well-known Δ^24^ inhibitor of 24-sterol methyl transferase (24-SMT), an enzyme that plays an essential role in the parasite sterol biosynthesis absent in mammalian host cells.^79^ The IC_50_ values for **H3** and **36** were 5.2 and 2.5 μM for promastigotes and 543 and 32 nM for intracellular amastigotes, respectively. The coordination of azasterol **H3** to the Zn(II) ion clearly increased the activity against both parasite developmental stages. Also, **36** proved to be 17 times more potent than **H3** against intracellular amastigotes. The more selective nature of **36** (SI = 156) than that of **H3** (SI = 20) was determined in cytotoxicity assays. Both **H3** and **36** were able to inhibit ergosterol biosynthesis of the *L. amazonensis* promastigotes. However, **36** promoted a 20% higher cholesterol accumulation compared to **H3**. Both compounds could inhibit 24-SMT by the depletion of endogenous sterols in the parasite cells. Moreover, ROS production and mitochondrial membrane potential analysis confirmed significant ultrastructural changes in the parasite. The structural lesions in the endoplasmic reticulum and mitochondrion resulted in a significant increase in ROS production and a decrease in the mitochondrial membrane potential.^78^ These results suggest at least two different mechanisms of action for **36**, which could explain its superior potency to that of **H3**.

## 4 Zinc Complexes against Chagas Diseases

Trypanosomiasis, more commonly known as Chagas disease, is a parasitic disease prevalent in Latin America and Central American countries. It is estimated that approximately 6–7 million people are infected worldwide, with a significant portion of cases concentrated in Brazil.^80^ Chagas disease is caused by the protozoan *Trypanosoma* (*T.*) *cruzi* and is considered the most lethal parasitic disease in Latin America, responsible for about 10,000 deaths annually. The primary mode of transmission is the bite of blood-sucking insects infected with the parasite, belonging to the species *Triatoma infestans*, commonly known as kissing bugs. The disease can also be transmitted via other routes, congenital (from mother to child), transfusional (through blood transfusions or blood products), accidental (in laboratories), and organ transplantation.^81^

**Figure 12:**
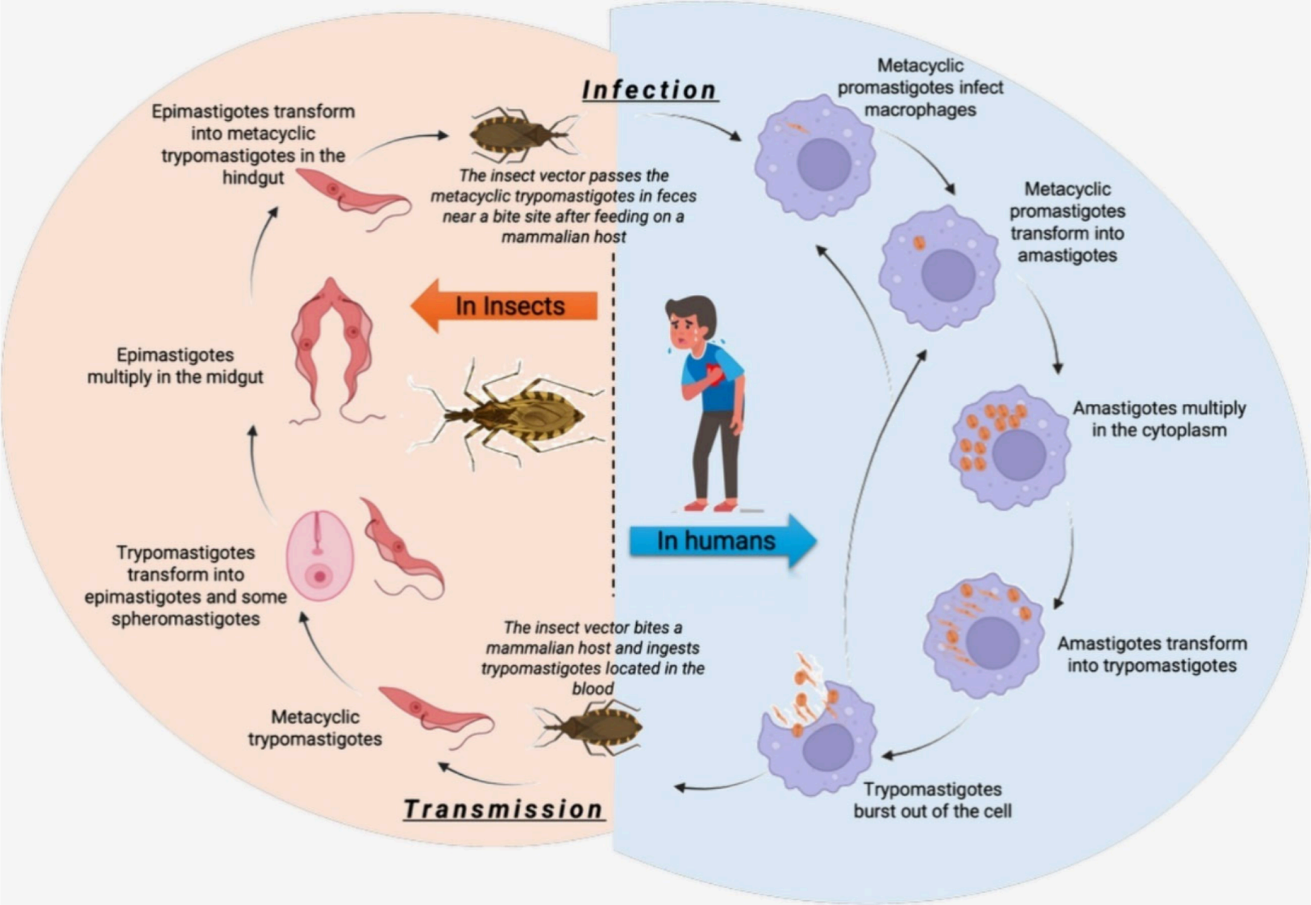
Life cycle of *Trypanosoma cruzi* (Chagas disease).

Chagas disease presents in two forms: acute and chronic. The acute phase may either be asymptomatic or exhibit nonspecific symptoms like myocarditis, malaise, fever, headache, and loss of appetite. Left untreated, the infection progresses to the chronic phase, posing risks of severe cardiac and digestive complications and potentially leading to death. The Chagas disease cycle (Figure 12) involves multiple stages in both the human host and the vector. It initiates when an infected kissing bug feeds on a person’s blood, leaving behind feces containing *T. cruzi*, the disease-causing agent. These infective forms enter the human body through bite wounds, mucous membranes, or damaged skin.^82^ The life cycle of *T. cruzi* involves several stages: the insect vector (either female or male) injects metacyclic trypomastigote form in the feces, which then infects macrophages in the mammalian host, and inside, they transform into amastigotes. Amastigotes are released from the parasitophorous vacuole and multiply in the cytoplasm of the host cell. They then transform back into trypomastigotes, which burst out of the host cell. Both amastigotes and trypomastigotes are formed and go on to infect new macrophages. In the transmission process, the vector bites a mammalian host and ingests (metacyclic) trypomastigotes from the blood. Within the insect, trypomastigotes transform into epimastigotes and some spheromastigotes. The former multiplies in the midgut of the insect, while the latter transforms into metacyclic trypomastigotes in the hindgut.

In the acute phase, infective forms transform into amastigotes near the entry site, they replicate, and they revert to trypomastigotes, spreading by breaching host cells and entering the bloodstream to infect other cells. Healthy kissing bugs contract infection by biting infected humans and ingesting blood containing trypomastigotes. Inside the bug’s gut, trypomastigotes morph into epimastigotes, multiply, and migrate to the hindgut, where they differentiate into infective forms, poised to infect another human host.^83^

**Figure 13:**
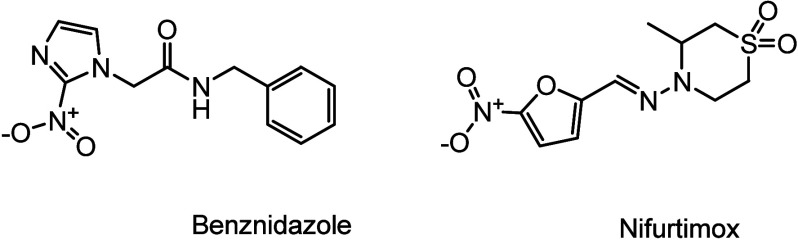
Chemical structures of benznidazole and nifurtimox.

Currently, the main treatment options for Chagas disease are benznidazole and nifurtimox (Figure 13). However, the use of nifurtimox was discontinued in Brazil in the late 1980s due to its severe, often intolerable side effects, such as neurosensory and gastrointestinal effects. Although these drugs demonstrated high efficacy when administered during the acute stage of the disease, their effectiveness is limited in the chronic phase. Additionally, the treatment is prolonged, often lasting weeks to months, which can cause significant adverse effects.^84,85^

Despite advances in research, a vaccine against the Chagas disease is not yet available. Attempts faced significant challenges due to the complexity of the *T. cruzi* parasite and its interaction with the host’s immune system. Despite these efforts, more effective and less toxic drugs have not been identified. The focus of the research is to find new therapies that can improve clinical outcomes and the quality of life of patients with Chagas disease^86^ as shown below.

The zinc complex [Zn(Fctpy)_2_](PF_6_)_2_ mentioned earlier (**11**, Figure 5) showed activity against the epimastigote form of *T. cruzi*, with IC_50_ = 52.5 μM. This demonstrated the potential of this zinc compound as a potential therapeutic agent against *T. cruzi* in addition to its antimalarial activity, broadening its application in the field of infectious diseases.^41^

**Figure 14:**
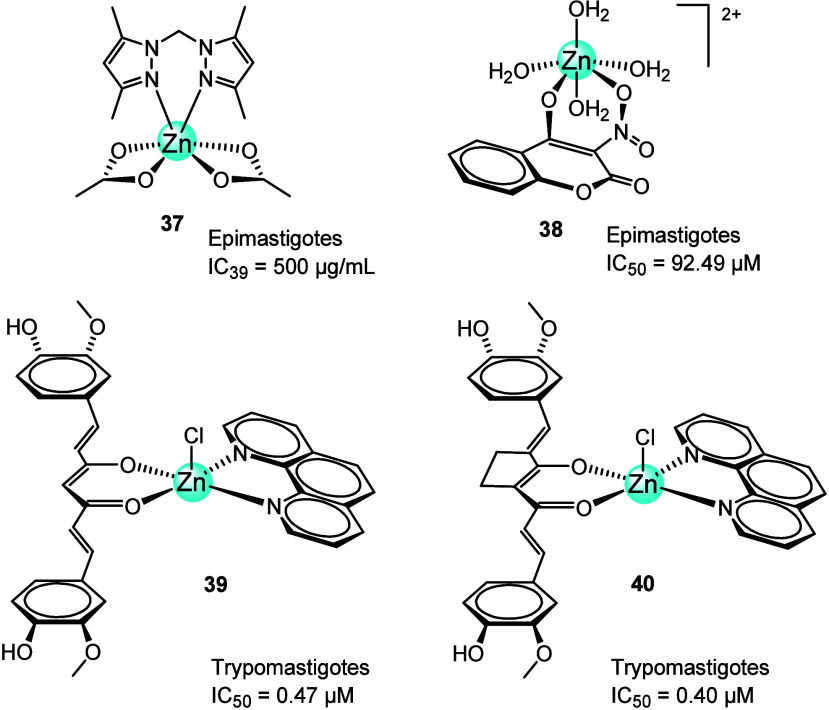
Chemical structures of Zn(II) containing bidentate ligands (**37**–**40**) tested against *T. cruzi*.

Hurtado et al.^87^ in 2019 reported the synthesis and characterization of seven complexes of Zn(II), Cu(II), Co(II), and Ni(II) and ligands derived from bis(3,5-dimethylpyrazol-1-yl)methane (bdmpzm) and bis(3,5-dimethyl-4-nitro-1*H*-pyrazolyl)methane. The biological evaluation of the complexes and their respective ligands against epimastigotes of *T. cruzi* revealed an enhancement in activity compared to the free ligands, with IC_50_ values ranging from 45.7 to 176 μg/mL. Focusing on the Zn(II) complex (**37**, Figure 14), the data indicated low activity against *T. cruzi*, presenting 39% inhibition at a high concentration of 500 μg/mL.^87^

De Alcantara et al.^88^ used coumarin (4H3NC), a secondary metabolite found in numerous plants to create metal complexes, including zinc (4H3NCZn, **38**, Figure 14). All metal complexes were evaluated *in vitro* against two strains of *T. cruzi* epimastigotes. Results showed that **38** was less effective (IC_50_ 92.49 μM) than other metal complexes: for example, when compared with the Fe complex (4H3NCFe, IC_50_ = 21.55 μM) and benznidazole as the reference drug (IC_50_ = 36.10 μM). In general, the presence of metal ions (Zn(II) or Fe(II)) enhanced the trypanocidal activity, probably due to changes in the electron density of 4H3NC (IC_50_ = 332.20 μM) caused by the coordination of metal ions.

Neves and colleagues^89^ also prepared two zinc-curcuminoid-based complexes containing curcumin (CU) and its carbocyclic analog (CU-A), Zn(CU)(phen)Cl (**39**, Figure 14) and Zn(CU-A)(phen)Cl (**40**, Figure 14), and evaluated their activity against *T. cruzi* epimastigotes. Both **39** (IC_50_ = 0.47 μM) and **40** (IC_50_ = 0.40 μM) were more effective than copper analogs and benznidazole (IC_50_ = 10.3 μM). However, they were significantly less selective than benznidazole (SI = 4.3, 3.8, and 70 for **39**, **40**, and the reference drug, respectively). It is worth noting that the phenanthroline ligand contributes to the enhanced trypanocidal activity of these Zn(II)-compounds.

As mentioned before, Navarro and co-workers^76^ synthesized two Zn(II) complexes, [Zn(ITZ)_2_Cl_2_] (**32**, Figure 10) and [Zn(ITZ)_2_(OH)_2_] (**33**, Figure 10), which exhibited a broad spectrum of action with antiparasitic and antifungal activity at nanomolar concentrations. These compounds **32** and **33** inhibited the proliferation of the epimastigote forms of *T. cruzi* with IC_50_ 3.31 and 1.3 nM, respectively, and the amastigote forms with IC_50_ 1.14 and 0.91 nM, respectively. Both Zn-ITZ complexes were more active than the ligand alone (ITZ), with **32** being the most active (Figure 11).

Salas, Cabarello, Méndez-Arriaga, et al.^62−71^ evaluated the antileishmanial activity of several zinc(II) complexes as well as **20** and **22** (Figure 9) against *T. cruzi*.^68,69,71^ [Zn(dmtp)_2_(H_2_O)_4_](ClO_4_)_2_·2dmtp·2H_2_O (**20**) inhibited the proliferation of amastigote forms of *T. cruzi* with an IC_50_ value of 19.2 μM, similar to that of benznidazole (IC_50_ 15.8 μM),^69^ while [Zn(ftpO)_2_(H_2_O)_4_] (**22**) was less active with IC_50_ > 200 μM.

**Figure 15:**
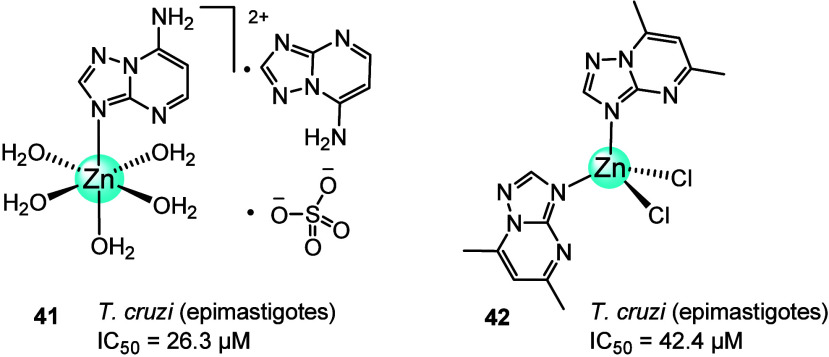
Active Zn(II) triazolopyrimidine complexes (**41** and **42**) against the epimastigote form of *T. cruzi*.

In 2022, a series of Zn complexes of triazolopyrimidine derivatives were synthesized with Zn(SO_4_)·7H_2_O, ZnCl_2_, and Zn(NO_3_)_2_·6H_2_O salts. The antiparasitic activity of [Zn(7atp)(H_2_O)_5_](7atp)(SO_4_) (**41**) and [ZnCl_2_(dmtp)_2_] (**42**) (Figure 15) together with **19**–**21 (**previously discussed against *Leishmania* parasites) was tested *in vitro* after 72 h of incubation against *T. cruzi* to evaluate their effect on Chagas’ disease. The cytotoxicity against the epimastigote form of these zinc complexes was lower than that of the reference drug, with IC_50_ values between 25.4 and 65.6 μM. Despite this, all Zn complexes had an improved selectivity index compared to the free ligands and the reference drug, which demonstrated their synergistic behavior in antiparasitic activity.^90^

**Figure 16:**
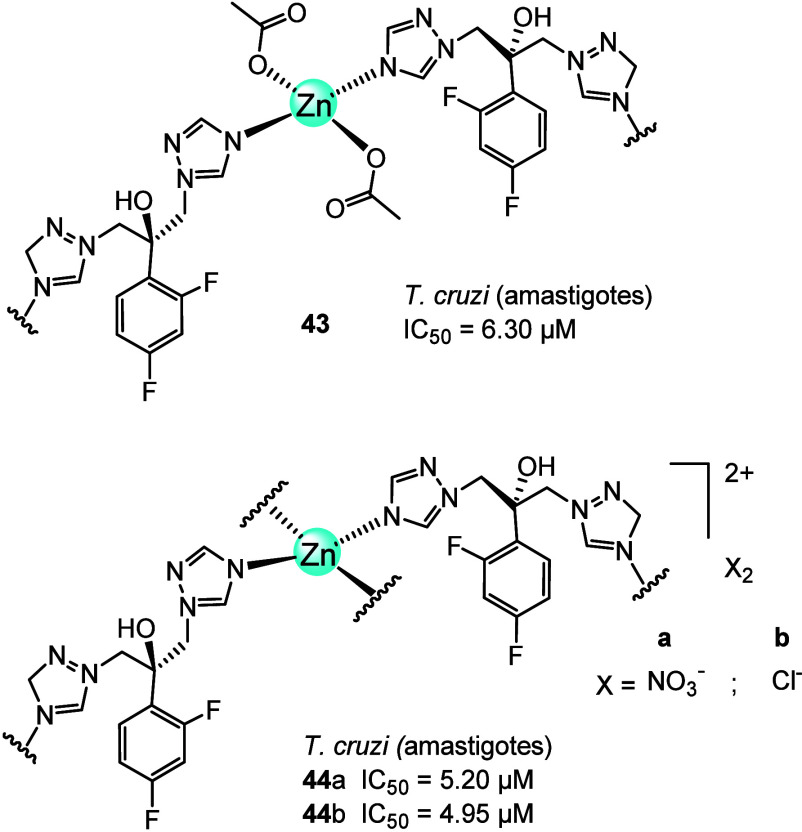
Chemical structures of fluconazole-based Zn(II) coordination polymers.

Then, the Navarro group^91^ synthesized a series of transition metal coordination polymers, including zinc (**43**, **44a**, and **44b**, Figure 16) through the reaction of fluconazole (FLZ) with zinc salts. The anti-Chagas activity of these compounds was evaluated against various forms of *Trypanosoma cruzi*; however, results for only the clinically relevant, amastigote stage of *T. cruzi* were presented. IC_50_ for **43**, **44a**, and **44b** were 6.30, 5.20, and 4.95 μM, respectively, lower than that of the free FLZ (IC_50_ = 24.3 μM). Significant ultrastructural changes induced by **44b** in epimastigotes and intracellular amastigotes were confirmed by TEM, which could lead to parasite death. Similar to the Zn-ITZ complexes discussed above, the strategy of combining zinc with FLZ proved to be effective in increasing activity against *T. cruzi*. There is potential for future applications of these metal-FLZ coordination polymers in the treatment of parasitic diseases (Figure 16).

**Figure 17:**
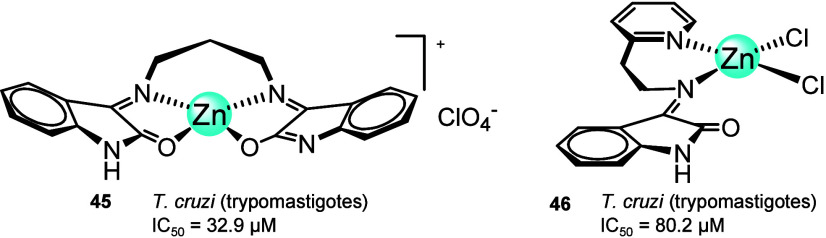
Chemical structures of oxindolimine-Zn(II) complexes.

More recently, in 2023, Da Costa Ferreira et al.^92^ selected two zinc(II) complexes of oxindolimine (**45** and **46**, Figure 17) to determine their biological activity against the infective trypomastigote forms and the intracellular amastigote forms of *T. cruzi*. This was intriguing, as their activity was known for inhibiting the proliferation of tumor cells, having the DNA and the mitochondria as main targets through an oxidative mechanism causing apoptosis.

Complexes **45** and **46** induced the trypomastigotes forming with IC_50_ = 32.9 and 80.2 μM at 24 h and 11.3 and 56.2 μM at 48 h, respectively. The SI of **45** after 48 h of incubation was 12.4, higher than that of **46 (**SI = 2.7). When tested against amastigotes, the infection rate was less than 10% after 24 or 48 h in the presence of either zinc(II) complex (60–80 μM). The authors concluded that further studies are needed to elucidate the probable modes of action and to develop other, more effective compounds as trypanocidal agents.^92^

## 5 Zinc Complexes against African Trypanosomiasis

The parasitic illness known as African Trypanosomiasis, Sleeping Sickness, or “Old World Trypanosomes” is caused by *Trypanosoma* protozoa. Tsetse flies (Glossina species), which get the parasites from infected people or animals, spread the infection with their bite. The illness is endemic to Sub-Saharan Africa and is typically lethal if left untreated. Those who live in rural areas and work in agriculture, fishing, cattle, or hunting are the most vulnerable.

There are three ways that African trypanosomiasis presents itself, based on the subspecies of the parasite.

- *T. brucei gambiense*, often known as *Trypanosoma brucei*, found in 24 West and Central African nations, makes up 92% of reported cases. It causes a chronic illness for which a person may be sick for months or years without experiencing any noticeable symptoms. Frequently, the disease has progressed and compromised the central nervous system by the time symptoms manifest.
- *T. brucei rhodesiense*, found in 13 East and Southern African nations, makes up 8% of the reported cases. It causes an acute illness, exhibiting symptoms a few weeks or months after infection. The illness spreads quickly and affects several organs, including the brain.
- The third subspecies, *T. brucei brucei*, mainly attacks cattle and sometimes other animals, but it typically does not infect humans under normal circumstances.

The number of new cases of *T. brucei* has decreased by 98% between 1999 and 2023, down to 675 from 27,862. Similarly, the number of new cases of *T. brucei rhodesiense* decreased by 96% from 619 to 24 during the same period.^93^

**Figure 18:**
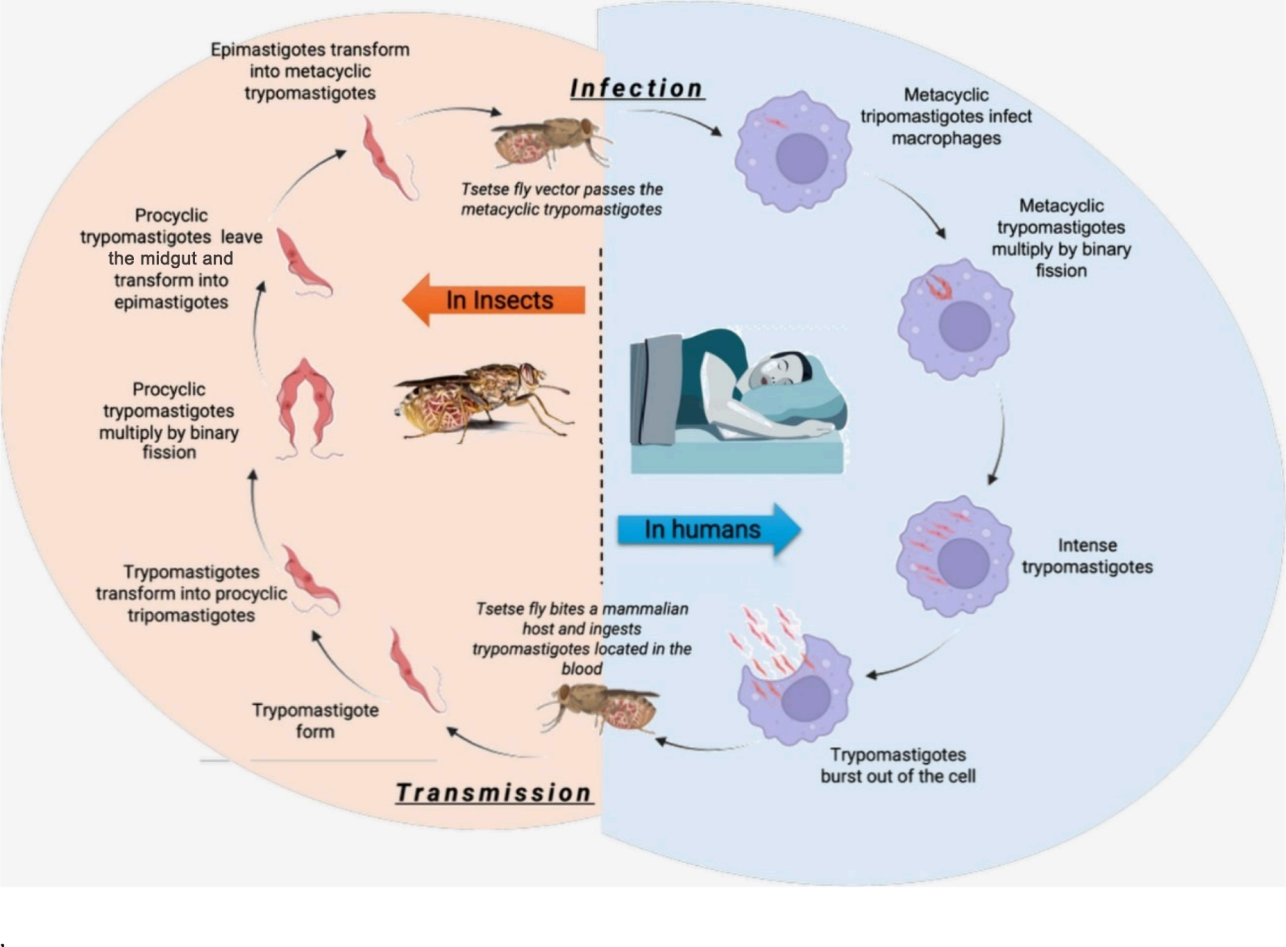
Life cycle of the *Trypanosoma brucei* parasite (sleeping sickness).

In the cycle life of the parasite represented in Figure 18, during the bite of a mammalian host, an infected tsetse fly injects metacyclic trypomastigotes into the skin tissue. These parasites then enter the lymphatic system and, subsequently, the bloodstream. Inside the host, they transform into bloodstream trypomastigotes, which are distributed to various parts of the body, including other body fluids such as lymph and spinal fluid, where they continue to replicate through binary fission. The entire life cycle consists of extracellular stages. Another tsetse fly becomes infected with bloodstream trypomastigotes when it feeds on an infected mammalian host. In the fly’s midgut, the parasites transform into procyclic trypomastigotes, multiply by binary fission, exit the midgut, and transform into epimastigotes. These then migrate to the fly’s salivary glands, where they continue to multiply by binary fission. The cycle within the fly takes about 3 weeks. On rare occasions, *T. b. gambiense* can be transmitted congenitally if the mother is infected during pregnancy.^94^

**Figure 19:**
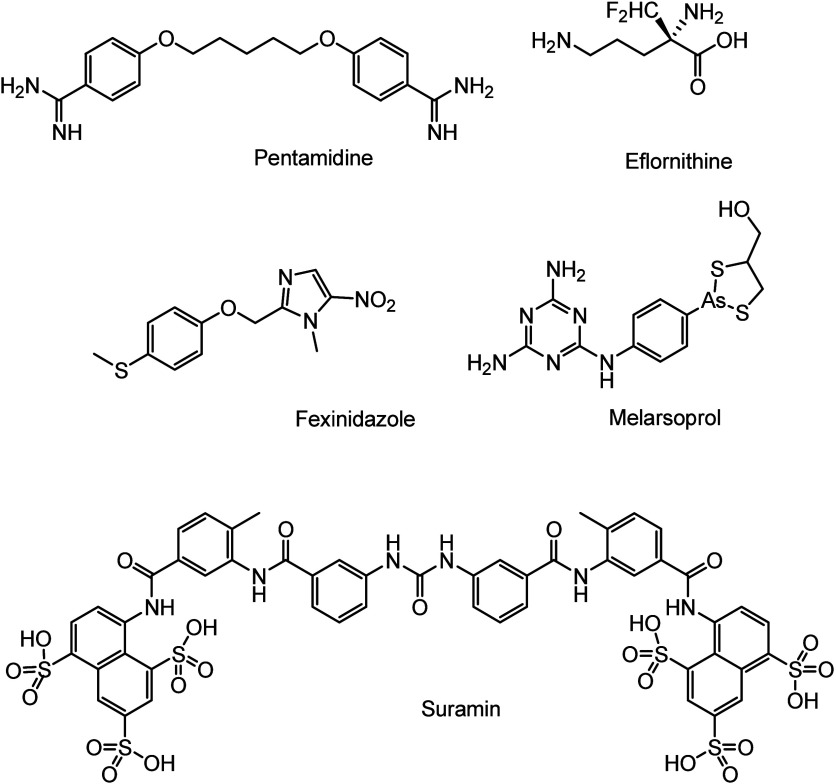
Chemical structures of common medications used against African trypanosomiasis.

Treatment options depend on strain and disease stage. The four common treatments available for *T. brucei* include pentamidine, which is given intramuscularly in the initial phase and is well tolerated, eflornithine, administered intravenously, used as monotherapy or in combination with nifurtimox (also available as monotherapy); both options are often used in the second disease stage. Oral fexinidazole is used in both stages. For *T. brucei rhodesiense* infection, intravenous suramin is used in the first stage and melarsoprol in the second stage, despite its serious side effects, including encephalopathy, which can be fatal.^95^ The structures of these drugs are shown in Figure 19.

Metal complexes against *T. brucei* are studied significantly less extensively than other diseases, and the number with zinc as the central metal is especially limited. This may be partly attributed to the decrease in the number of new cases in recent years. However, research for new treatment options remains essential, as there are currently 57 million people at risk of contracting this disease in 36 countries.^96,97^

**Figure 20:**
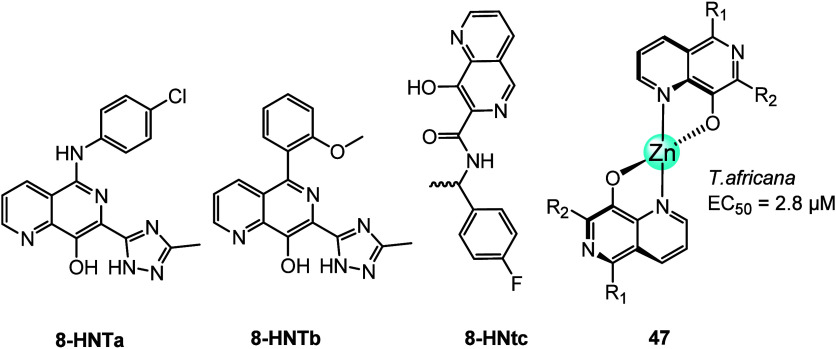
Chemical structures of three 8-HNT compounds and zinc complex **47**.

In 2018, Wyllie and co-workers investigated the impact of zinc on the potency of 8-hydroxy-naphthyridines (8-HNT) (Figure 20) against *T. brucei*. They found that the depletion of zinc import to the Golgi apparatus reduced the efficacy of these compounds. It was hypothesized that 8-HNT lowering zinc levels in trypanosomes and increased levels in cytoplasm could reduce their potency. To test this theory, exogenous zinc was added to the parasites. The addition of ZnCl_2_ at 200 μM did not affect cell growth and viability but decreased the potency of 8-HNTa by 8-fold, EC_50_ increased from 0.36 μM to 2.8 μM. Similar zinc-dependent potency drops were observed with all three 8-HNTs. Ultimately, the authors concluded that the cytotoxic effects of 8-HNT are due to the chelation of bivalent cations, suggesting that the active species against *T. brucei* is the zinc complex of 8-HNT (**47**, Figure 20).^98^

**Figure 21:**
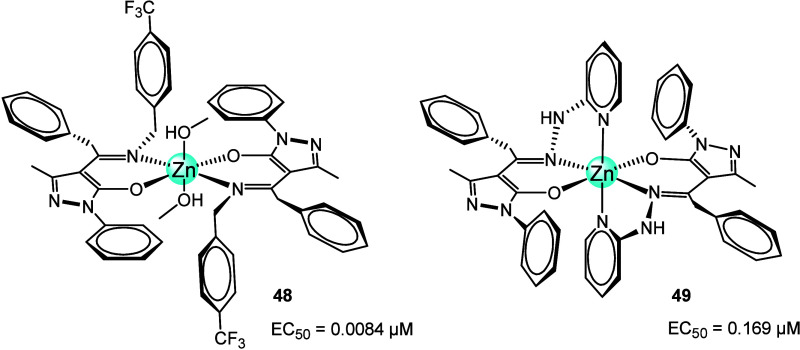
Zinc complexes **48** and **49** with pyrazolone-based hydrazones with activity against *T. brucei*.

In 2022, Marcheti et al. introduced two novel pyrazolone-based hydrazones and their Zn^2+^ complexes, which were fully characterized and investigated as potential antitrypanosomal agents. Notably, complexes **48** and **49** (Figure 21) both demonstrated significant antitrypanosomal activity against *T. brucei* in *in vitro* studies, and very low cytotoxicity, with EC_50_ 0.0084 μM (**48**) and 0.169 μM (**49**).^99^ The mechanism of action was elucidated through the analysis of nucleoside triphosphates (NTP and dNTP pools) by HPLC, including CTP (cytosine triphosphate), UTP (uridine triphosphate), ATP (adenosine triphosphate), and GTP (guanine triphosphate). These molecules are essential for various cellular functions such as RNA synthesis, phospholipid production (CTP), protein glycosylation (UTP), the primary energy source (ATP), and cellular signaling via G-proteins (GTP). The analysis of the NTP pool in the parasites provides insights into their metabolic state, the availability of precursors for RNA and DNA synthesis, and the effectiveness of drugs that may interfere with these processes. The free ligand H2L1 was inactive, but complex **48** targeted the enzyme CTPS which impacted the CTP levels. Its effect might be due to the chelation of naturally occurring metals in the trypanosomes or growth medium. This finding is significant as *T. brucei* does not have alternative pathways for CTP synthesis, which makes CTPS a critical target.

## 6 Conclusion

Parasitic infectious diseases, such as malaria, leishmaniasis, Chagas disease, and African trypanosomiasis (sleeping sickness), have long affected millions of people globally, particularly in poor countries. The treatment would allow those affected to live full, healthy lives everyone has basic human rights to.

The first part of this review was a general description as to why zinc may be a metal that is well suited for use as a potential therapeutic antiparasitic agent.

We then turned our attention to malaria, the different control strategies and their challenges, and the available vaccines and treatments. It is well-known that monotherapies are no longer used and are replaced by combinations of two or more drugs. However, these combination therapies have several limitations and cannot eradicate malaria; which, in fact, still kills one child every minute. Therefore, the development of metallodrugs for the well-designed prevention of transmission and treatment of this deadly disease is pressing. Zinc complexes can be an excellent choice for effective, multitarget antimalarial metallodrugs.

The zinc complexes of known antimalarial drugs yielded more effective compounds based on their synergistic effect. Those containing different ligands also showed potent activity against chloroquine-sensitive and chloroquine-resistant strains *in vitro*, some in the nanomolar concentration range. The Zn(II) complexes were able to interact with several essential parasite targets, such as the inhibition of β-hematin, DNA, enzyme purine nucleoside phosphorylase in *P. falciparum*, and cysteine protease enzymes falcipain-2 and falcipain-3. Some complexes have also been evaluated *in vivo*.

The review also summarizes the reported articles on zinc complexes against neglected diseases such as leishmaniasis, Chagas disease, and African trypanosomiasis. These diseases are considered neglected because there are no available vaccines, the number of drugs is minimal, their efficacy and safety are far from ideal, and to make matters worse, the parasite is developing resistance to some of these drugs. The efficacy of the zinc complexes was tested *in vitro* against different protozoan parasites. Some demonstrated efficacy at low micromolar concentrations, outperforming traditional drugs in certain cases. They often showed lower toxicity to host cells compared to the reference drugs, indicating a potentially better safety profile. The most promising Zn complexes are Zn-ITZ and ZnCl_2_(H_3_)_2_, which inhibited the proliferation of the amastigote forms of *T. cruzi* and *L. amazonensis* in the nanomolar range and had remarkable selectivity. The latter acts as an inhibitor of ergosterol biosynthesis of the *Leishmania* parasite. According to our review, few Zn complexes have been tested *in vivo* against these neglected diseases.

The promising results achieved with Zn complexes against the parasites that cause malaria, Leishmania, Chagas disease, and sleeping sickness encourage a detailed, in-depth study of the mechanism of action to identify the essential parasite target(s) in the hope of developing more effective and safer antiparasitic treatments.

This review aimed to demonstrate the real potential of zinc complexes in the development of rational, effective, and safe drugs against protozoa that cause parasitic infections. The available, remarkable scientific data on the antiparasitic activity and selectivity of those zinc complexes may warrant further experiments such as preclinical and clinical studies, which could lead to chemotherapy to save the millions of ill people suffering from these parasitic diseases.
